# Identification and validation of a prognostic signature comprising inflammation and pyroptosis-related genes in oral squamous cell carcinoma

**DOI:** 10.3389/fimmu.2026.1721849

**Published:** 2026-07-07

**Authors:** Junfu Wu, Jie Fan, Shengli Shao, Wenhui Shen, Gang Li, Shanting Liu, Wei Du

**Affiliations:** Department of Head and Neck Surgery, The Affiliated Cancer Hospital of Zhengzhou University & Henan Cancer Hospital, Zhengzhou, China

**Keywords:** inflammation, prognosis, pyroptosis, risk assessment, squamous cell carcinoma of head and neck

## Abstract

**Background:**

Oral squamous cell carcinoma (OSCC) represents a common malignancy characterized by significant morbidity and mortality rates, highlighting the critical necessity for novel therapeutic approaches. Consequently, investigating differentially expressed genes linked to inflammation and pyroptosis may identify potential prognostic biomarkers and therapeutic targets.

**Methods:**

To address this research gap, our study utilized an extensive bioinformatics approach by analyzing the Cancer Genome Atlas Head and Neck Squamous Cell Carcinoma (TCGA-HNSC) dataset through differential expression analysis to identify genomic features associated with OSCC. Subsequent analyses included Gene Ontology and pathway enrichment assessments, along with survival analyses using Cox regression models, to evaluate the prognostic significance of the identified differentially expressed genes. Furthermore, immune infiltration analysis and somatic mutation assessments were conducted to elucidate the relationship between immune cell types and key prognostic genes. Additionally, copy number variation analysis was performed to highlight genomic alterations associated with immune-related and prognostic-related differentially expressed genes(DEGs).

**Results:**

Our analysis identified a total of 3,495 differentially expressed genes, among which 53 immune-related and prognosis-related differentially expressed genes demonstrated a significant correlation with the prognosis of OSCC. Immune infiltration analysis further revealed the presence of 28 immune cell types within OSCC samples, with a notable prevalence of activated CD8 T cells and regulatory T cells, underscoring their association with critical prognostic genes. Additionally, pathway analysis highlighted the activation of cytokine signaling pathways and their associations with processes relevant to systemic lupus erythematosus. A prognostic risk model derived from these findings effectively stratified patients based on overall survival, identifying four key genes—*CTSG,HKDC1,PTX3* and *SPP1*—as crucial prognostic indicators. This analysis may uncover potential prognostic biomarkers and therapeutic targets.

**Conclusion:**

This study established a novel OSCC prognostic risk model based on inflammation- and pyroptosis-related interactive genes. *CTSG, HKDC1, PTX3* and *SPP1* were validated as independent prognostic biomarkers, and the risk score was closely associated with tumor microenvironment features, metabolic activity, and therapeutic sensitivity. This work may provides substantial references for the exploration of novel biomarkers for OSCC treatment and facilitates clinical decision-making.

## 1 Introduction

Oral squamous cell carcinoma (OSCC) is one of the most common malignancies in the head and neck region, with a steadily increasing global incidence. According to the 2022 global cancer statistics, there were 389,000 new cases and more than 188,000 deaths of oral cancer worldwide (1), including 65,000 new cases and 35,200 deaths in China (2). OSCC severely impairs patients’ quality of life and imposes a heavy economic burden on the public healthcare system (3). Although conventional therapeutic approaches, including surgical resection, radiotherapy, and chemotherapy, have been well developed, the overall prognosis of OSCC patients remains poor due to the high invasiveness and recurrence rate of tumors (4). Therefore, exploring novel biomarkers and therapeutic targets to optimize current diagnostic and therapeutic strategies is essential to address the clinical challenges of OSCC.

Regulated cell death constitutes a core biological framework for elucidating the progression and therapeutic responses of OSCC, and the regulatory roles of inflammation and pyroptosis have become research hotspots in oncology (5). Pyroptosis is an inflammatory form of programmed cell death characterized by inflammasome and caspase activation, gasdermin-mediated plasma membrane pore formation, and the release of pro-inflammatory cytokines including interleukin-1β and interleukin-18, which distinctly differentiates it from nonspecific and passive cell necrosis. Moreover, pyroptosis exhibits unique mechanistic features compared with other regulated cell death modalities. It induces stronger inflammatory cytokine release than apoptosis and strictly relies on gasdermin-mediated membrane pore formation, which distinguishes it from necroptosis. In contrast, ferroptosis and autophagy-dependent cell death are driven by iron-dependent lipid peroxidation and excessive activation of intracellular degradation systems, respectively (5–7). Accumulating evidence has demonstrated that various regulated cell death pathways are not independent. Upstream events such as inflammatory activation, oxidative stress, immune checkpoint dysfunction, and metabolic reprogramming mediate extensive crosstalk among different signaling pathways, thereby modulating tumor immune clearance and therapeutic sensitivity (8). The emerging concept of PANoptosis further confirms shared molecular execution mechanisms among pyroptosis, apoptosis, and necroptosis, providing novel theoretical evidence for explaining the complex relationships among cell death, inflammatory responses, and immune evasion in OSCC (7). In the OSCC tumor microenvironment, inflammation and pyroptosis form a tight bidirectional regulatory network. Inflammatory signals modulate the activation of pyroptotic pathways, while pyroptosis further amplifies local inflammatory responses and reshapes tumor immune infiltration, ultimately regulating OSCC progression and patient prognosis (5). Given the close biological interaction between inflammation and pyroptosis, individual analysis of inflammation-related or pyroptosis-related genes cannot fully reveal their synergistic regulatory effects. Combined analysis of the two gene sets enables the precise screening of candidate genes closely associated with OSCC progression and prognosis from the perspective of crosstalk between inflammatory activation and pyroptosis regulation, and helps explore the intrinsic relationships between these key molecules, patient prognosis, and immunotherapy responsiveness, providing new insights for mechanistic research and therapeutic optimization in OSCC.

Based on the above research background, this study integrated public transcriptomic datasets and inflammation- and pyroptosis-related gene sets to screen core prognostic genes of OSCC. A prognostic risk model for OSCC was subsequently constructed and systematically validated, aiming to provide a reliable theoretical reference for prognostic evaluation and targeted clinical therapy of OSCC.

## 2 Materials and methods

### 2.1 Data download

Data of head and neck oral squamous cell carcinoma (HNOSCC) were obtained from The Cancer Genome Atlas (TCGA) using the TCGAbiolinks R package (9). After excluding samples without clinical data, 329 OSCC samples and 32 normal controls were retained and normalized to fragments per kilobase of transcript per million mapped reads. Clinical data were retrieved from the UCSC Xena database (10) (https://xena.ucsc.edu/). For validation, datasets GSE23558 (11), GSE25099 (12), and GSE41613 (13) from human oral tissues were acquired via the GEOquery R package. These include GSE23558 (27 OSCC, 5 normal), GSE25099 (57 OSCC, 22 normal), and GSE41613 (97 OSCC), with further details in **Supplementary Tables 1** and **S2**.

A total of 11,288 unique inflammation-related genes (IRGs) (**Supplementary Table 3** for details) were identified from GeneCards and MSigDB, while 534 unique pyroptosis-related genes (PRGs) (**Supplementary Table 4** for details)were compiled from the same sources. Of these, 513 genes overlapped between IRGs and PRGs (**Supplementary Table 5** for details). The datasets GSE23558 and GSE25099 were merged into a single GEO dataset with 84 OSCC and 27 normal samples using the sva R package. All datasets were normalized and standardized via the limma package (14), and the removal of batch effects was confirmed through PCA and box plots for the GSE41613 dataset.

### 2.2 Oral squamous cell carcinoma associated inflammation-related and pyroptosis-related differentially expressed genes

The TCGA-HNSC dataset was divided into OSCC and control groups for differential expression analysis using DESeq2 (14), with criteria of |logFC| > 2 and adj. P < 0.05 to identify differentially expressed genes (DEGs). Genes with logFC > 2 and adj. P < 0.05 were marked as upregulated, while those with logFC < -2 and adj. P < 0.05 were downregulated, using the Benjamini-Hochberg method for P-value adjustment. Results were visualized with a volcano plot via ggplot2. To identify OSCC-related inflammation and pyroptosis DEGs, DEGs meeting the criteria were intersected with inflammation- and pyroptosis-related DEGs, shown in a Venn diagram, and their expression profiles were displayed in heatmaps using pheatmap.

### 2.3 Somatic mutation copy number variation analysis

To investigate somatic mutations within the OSCC cohort of the TCGA-HNSC dataset, the “Masked Somatic Mutation” data was selected and preprocessed using VarScan software. Visualization was subsequently performed using the maftools R package (v2.18.0). For the analysis of copy number variations, the “Masked Copy Number Segment” data was utilized, processed, and analyzed employing GISTIC2.0 (15) with default parameters.

### 2.4 Gene ontology and pathway (KEGG) enrichment analysis

Gene Ontology (GO) and Kyoto Encyclopedia of Genes and Genomes (KEGG) enrichment analyses of IR&PRDEGs were conducted using the clusterProfiler R package (Version 4.10.0), applying criteria of adjusted P-value < 0.05 and False Discovery Rate (FDR) (Q value) < 0.25, with the Benjamini-Hochberg correction method.

### 2.5 Construction of inflammation and pyroptosis scores and weighted gene association network analysis

The single-sample Gene-set Enrichment Analysis (ssGSEA) method quantifies individual gene levels in datasets. Using the GSVA R package (v1.40.1), inflammation and pyroptosis scores were calculated for OSCC and control samples from the TCGA-HNSC dataset. The WGCNA R package was used to calculate gene correlation coefficients and construct a scale-free network, followed by hierarchical clustering to identify gene modules. The top 5000 variable genes from the OSCC group were selected, and parameters were set for module analysis. The correlation between inflammation, pyroptosis scores, and modules was assessed to identify signature genes. Genes with |R| > 0.3 were analyzed to map the relationship between inflammation and genetic variations.

### 2.6 Oral squamous cell carcinomas prognosis gene screening

We developed a prognostic risk model for OSCC using the TCGA-HNSC dataset. We applied Cox regression analysis with the R package survival (16) to evaluate the impact of Module Genes on prognosis. Variables with P< 0.1 from univariate analysis were selected for LASSO regression using the R package glmnet (v4.1-8) to prevent overfitting. Results were visualized with a prognostic risk model, variable trajectory plots, and a Forest Plot for multivariate analysis. Module Genes identified by LASSO were labeled as Prognostic Genes, and samples were divided into high-risk and low-risk groups based on the LASSO risk score, calculated as follows:


$$\text{risk}Score\;=\;\underset{i}{\sum }Coefficient(gene_{i})*mRNAExpression\;(gene_{i})$$

### 2.7 Prognostic analysis of OSCC

To investigate overall survival (OS) differences between the high-risk and low-risk OSCC groups within the TCGA-HNSC dataset, Kaplan-Meier (KM) curve analysis was conducted utilizing the R package ‘survival ‘ ((v3.5-7), grounded on LASSO risk scores. Furthermore, a time-dependent receiver operating characteristic (ROC) curve was constructed using the ‘survival ROC’ package to assess the model’s performance and the area under the curve (AUC) for predicting 1-, 3-, and 5-year survival outcomes. AUC values serve as indicators of diagnostic performance, with values exceeding 0.5 suggesting a propensity for event occurrence. Additionally, a risk factor Triple Plot was generated to depict OS differences and gene expression variations between the two risk groups.

### 2.8 Validation of prognostic risk model for Oral squamous cell carcinoma

To investigate the association between risk score and clinical variables, a univariate regression analysis was performed on risk score, age, gender, and clinical stage. Variables with significance levels of P < 0.10 were subsequently included in a multivariate Cox regression analysis, with results visualized through a Forest Plot. A Nomogram (17, 18) was developed using the R package rms (v6.7-1) to depict the relationship between risk score and clinical variables in terms of 1-, 3-, and 5-year survival outcomes. The Calibration Curve was employed to evaluate the model’s predictive accuracy by comparing actual outcomes to predicted probabilities derived from the Risk model.

### 2.9 Differential analysis of prognostic gene expression

To examine differences in prognostic gene expression between high-risk and low-risk groups within OSCC samples, group comparison plots were generated based on prognostic gene expression levels from the TCGA-HNSC and GSE41613 datasets. Additionally, to explore the variations in prognostic gene expression between the OSCC group and the control (normal) group, group comparison plots were also constructed using data from the TCGA-HNSC and the combined GEO dataset of GSE23558 and GSE25099.

### 2.10 Gene set enrichment analysis

GSEA analyzes gene distribution in predefined sets to evaluate their impact on phenotypes, comparing high-risk and low-risk OSCC groups from the TCGA-HNSC dataset. DESeq2 identified differentially expressed genes (logFC > 2, adj. P < 0.05), with up-regulated genes having logFC > 2 and down-regulated genes having logFC < -2, using Benjamini-Hochberg correction. Volcano and heatmaps of the top 20 DEGs were generated with ggplot2 and pheatmap. Genes were ranked by logFC, and GSEA was conducted with clusterProfiler (v4.10.0) using seed 2023, 10–500 genes per set, MSigDB c2 gene sets, and criteria of adj. P < 0.05 and FDR < 0.25.

### 2.11 Construction of protein-protein interaction (PPI) network and mRNA-miRNA regulatory network

The GeneMANIA database helps hypothesize gene functions, analyze gene lists, and prioritize genes for further study by identifying related genes from large genomics and proteomics datasets. It also predicts gene functions based on these interactions. MicroRNAs (miRNAs) are crucial in organism development by regulating multiple target genes. To explore the link between prognostic genes and miRNAs, we used the StarBase v3.0 database to identify relevant miRNAs and visualized the mRNA-miRNA regulatory network using Cytoscape software.

### 2.12 Immune infiltration analysis (ssGSEA)

The R package ggplot2 (19) visualizes expression differences and immune cell correlations between low risk and high risk groups, using Spearman’s algorithm. Results are shown in heatmaps and bubble plots. ssGSEA (20) enrichment scores indicate immune cell infiltration in TCGA-HNSC samples, forming an infiltration matrix. ggplot2 also compares immune cell expression between control and OSCC groups. Significant immune cells are identified and analyzed further, with correlations visualized using the pheatmap package. Correlations between Prognostic Genes and immune cells are also analyzed with Spearman’s method and displayed in a bubble plot via ggplot2.

### 2.13 Tumor immune dysfunction and exclusion, microsatellite instabilityand tumor mutation burdenanalysis

To obtain TIDE immunoscore results for OSCC samples from the TCGA-HNSC dataset, we performed a comprehensive analysis of gene expression data utilizing the TIDE platform (21), which enabled the prediction of treatment responses and survival outcomes. The TIDE immunoscore was determined by applying the Mann-Whitney U Test to compare T cell dysfunction and exclusion between high risk and low risk OSCC cohorts. Additionally, we acquired Tumor Mutational Burden (TMB) and Microsatellite Instability (MSI) data from cBioPortal and assessed intergroup differences in TMB and MSI scores using the Mann-Whitney U Test.

### 2.14 Cell culture and quantitative real time-PCR

Normal oral mucosal epithelial cells (HOMEpiC) and oral squamous cell carcinoma cells (CAL-27, SCC-25, SCC-9, HSC-1) from the Chinese Academy of Sciences were cultured at 37 °C with 5% CO_2_ and harvested at 70%-80% confluency. Total RNA was extracted using the RNeasy Mini Kit, and quality was checked with NanoDrop 2000. cDNA was synthesized using the PrimeScript™ RT Kit. qRT-PCR for four prognostic genes was conducted on a StepOnePlus™ system with a 20μL reaction mix. The cycling conditions included an initial cycle at 95 °C for 30s, followed by 40 cycles of 95 °C for 5s and 60 °C for 30s, with melting curve analysis. Relative expression was determined using the 2^-ΔΔCt method with GAPDH as a control. Primer sequences are in **Supplementary Table 6**.

### 2.15 Statistical analysis

Data processing and analysis were conducted using R software (version 4.2.2). Independent Student’s t-tests assessed the significance of continuous variable comparisons between two groups, while the Mann-Whitney U test was used for non-normally distributed variables. For comparisons involving three or more groups, the Kruskal-Wallis test was applied. Spearman correlation analysis evaluated correlation coefficients between molecules. All P-values were two-tailed, with a P-value below 0.05 considered significant unless specified otherwise.

## 3 Results

### 3.1 Technology roadmap

Flow Chart for the Comprehensive Analysis of inflammation-related and pyroptosis-related differentially expressed genes (IR&PRDEGs) (**Supplementary Figure 1**).

### 3.2 Merging of oral squamous cell carcinoma datasets

The sva package was used to remove batch effects from OSCC datasets GSE23558 and GSE25099, as confirmed by boxplots (**Supplementary Figures 2A, B**) and PCA plots (**Supplementary Figures 2C, D**). The merged data of GSE23558 and GSE25099 was applied to verify differentially expressed genes between tumor tissues and normal tissues.The GSE41613 dataset was normalized with the limma package, and boxplots (**Supplementary Figures 2E, F**) showed that expression differences between samples were minimized, indicating successful batch effect removal.The GSE41613 dataset was utilized for external validation of the prognostic model and expression comparison between high- and low-risk groups.

### 3.3 The differentially expressed genes related to OSCC inflammation and pyroptosis

**Figure 1:**
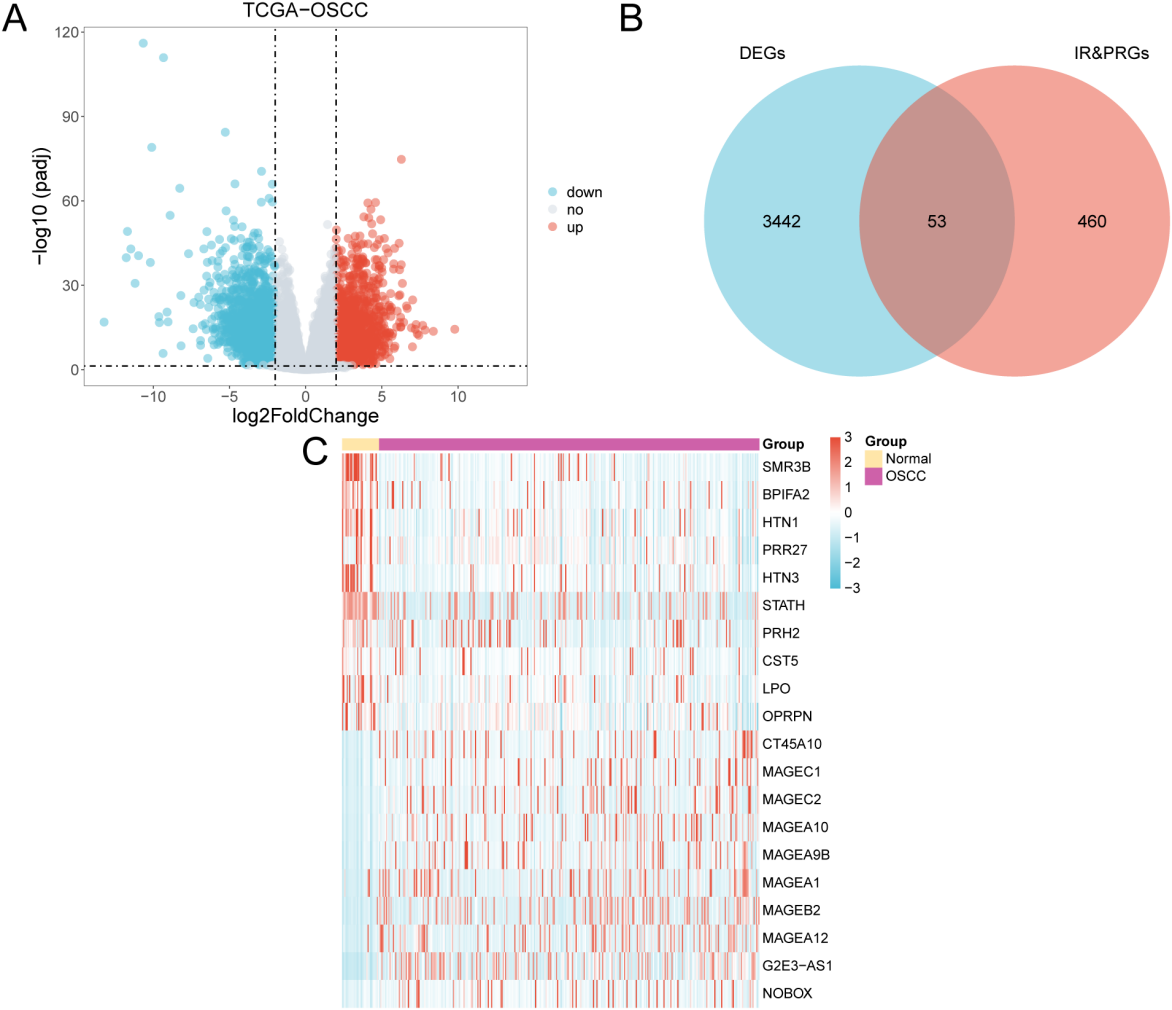
Differential gene expression analysis. **(A)** The volcano plot illustrates the analysis of differentially expressed genes (DEGs) between the oral squamous cell carcinoma (OSCC) group and the control (normal) group within the Head and Neck Squamous Cell Carcinoma dataset (TCGA-HNSC). **(B)** The DEGs and Venn diagram depict the intersection of inflammation-related and pyroptosis-related genes in the TCGA-HNSC dataset. **(C)** The heat map displays the inflammation-related and pyroptosis-related differentially expressed genes (IR&PRDEGs) within the TCGA-HNSC dataset. In this heat map, the OSCC group is represented in purple, while the control (normal) group is shown in yellow. Red indicates high expression levels, whereas blue indicates low expression levels.

Analysis of TCGA-HNSC data revealed 3,495 differentially expressed genes (DEGs), with 1,718 upregulated and 1,777 downregulated (**Figure 1A**). Among these, 53 inflammation and pyroptosis-related DEGs (IR&PRDEGs) met the criteria of |logFC| > 2 and adj.P < 0.05 (**Figure 1B**; details in **Supplementary Table 7**). A heatmap was created using the R package pheatmap to visualize expression differences across sample groups (**Figure 1C**).

### 3.4 Inflammation-related and pyroptosis-related differentially expressed genes, copy number variation, somatic mutation

**Figure 2:**
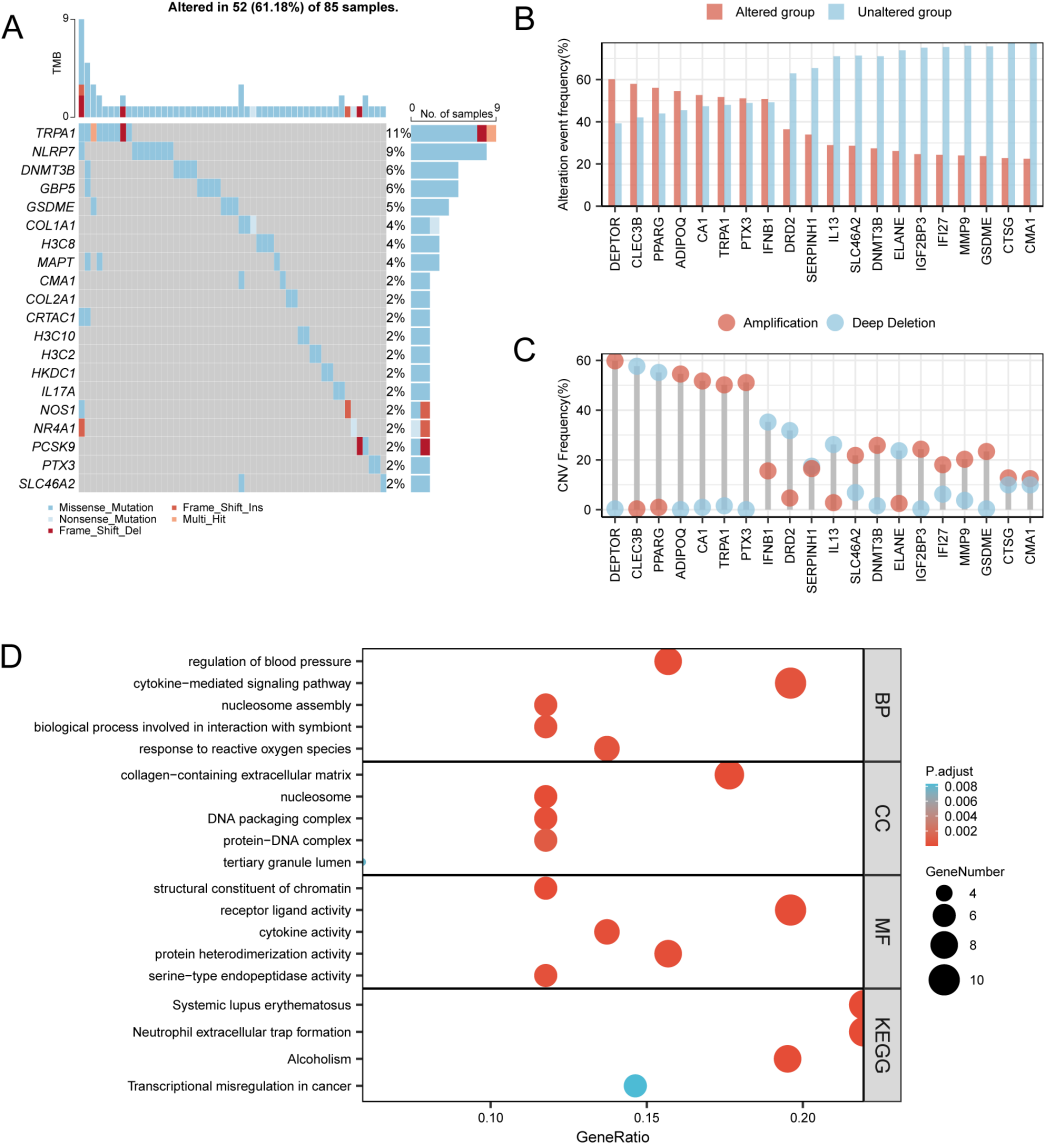
Somatic mutation (SM), copy number variation (CNV), GO and KEGG analysis. **(A)** SM of 53 inflammation- and pyroptosis-related differentially expressed genes (IR&PRDEGs) in OSCC were sorted by mutation frequency, and the top 20 mutated genes were visualized. **(B, C)**. The top 20 differentially expressed genes associated with inflammation and pyroptosis (IR&PRDEGs), exhibiting copy number variations (CNV), are identified in the OSCC cohort of the TCGA-HNSC. **(D)** The biological processes (BP), cellular components (CC), molecular functions (MF), and biological pathways (KEGG) are represented on the ordinate, alongside Gene Ontology (GO) and KEGG terms.

In the OSCC cohort derived from the HNSC dataset, SM of 53 IR&PRDEGs were categorized into four principal types, with missense mutations being the most prevalent. The mutations predominantly consisted of SNPs, with C-to-T transitions being the most frequent among SNVs. Among the top 20 genes ranked by mutation frequency, *TPRA1* exhibited the highest mutation rate at 11% (refer to **Figure 2A**). Furthermore, CNVs were identified in the 53 IR&PRDEGs within the OSCC group. The top 20 genes exhibiting the highest CNV frequencies included *DEPTOR, CLEC3B, PPARG, ADIPOQ, CA1, TRPA1, PTX3, IFNB1, DRD2, SERPINH1, IL13, SLC46A2, DNMT3B, ELANE, IGF2BP3, IFI27, MMP9, GSDME, CTSG*, and *CMA1* (see **Figures 2B, C**).

### 3.5 Gene ontology and pathway enrichment analysis

GO and KEGG analyses revealed that 53 IR&PRDEGs were predominantly enriched in biological processes such as blood pressure regulation, cytokine signaling, and pathways related to OSCC, including nucleosome assembly and the response to reactive oxygen species. These genes were linked to cellular components, such as the collagen-containing extracellular matrix, and molecular functions, such as receptor-ligand activity. Additionally, they were involved in pathways associated with systemic lupus erythematosus and transcriptional misregulation in cancer. The findings were visualized using bubble plots (**Figure 2D**; details in **Supplementary Table 8**).

Furthermore, a network diagram (**Supplementary Figure 3**) was developed based on the GO and KEGG results, illustrating biological processes (BP), cellular components (CC), molecular functions (MF), and KEGG pathways. The connections in the network diagram depict associated molecules and annotations, with node size representing the number of molecules perentry.

### 3.6 Construction of inflammation associated and pyroptosis score and weighted gene association network analysis

**Figure 3:**
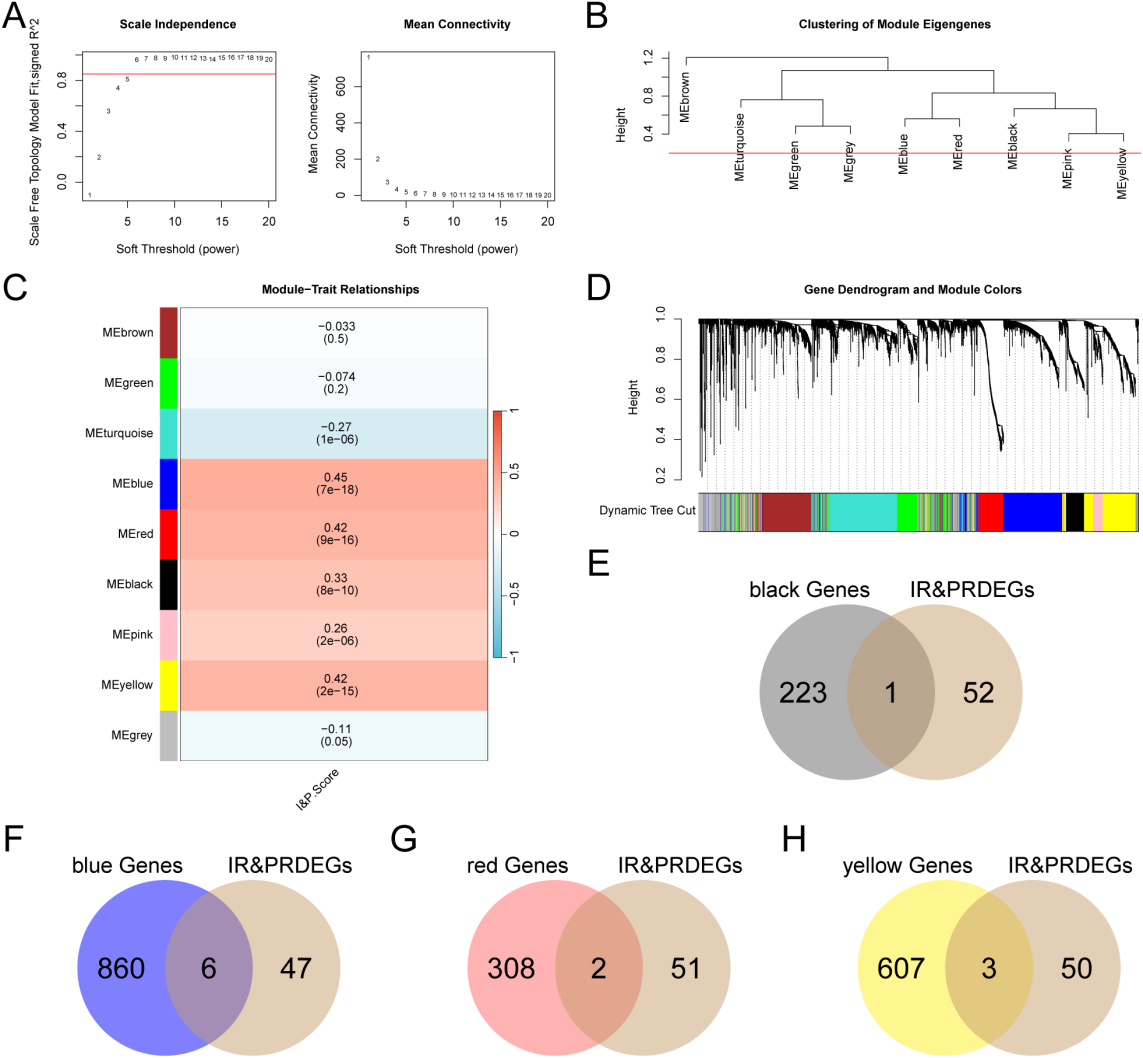
WGCNA for TCGA-HNSC. **(A)** The scale-free network shows the optimal soft threshold from WGCNA, with the left panel indicating the best threshold and the right panel illustrating network connectivity across various thresholds. **(B)** Gene module clustering results for the top 5000 variance genes. **(C)** Correlation analysis results between top 5000 variance gene modules and Inflammatory and pyroptosis scores. **(D)** Clustering results for top 5000 variance genes, with a hierarchical dendrogram above and gene modules below. **(E–H)** Venn diagrams of 53 inflammation and pyroptosis-related genes (IR&PRDEGs) with Black **(E)**, Blue **(F)**, Red **(G)**, and Yellow **(H)** modules. Correlation strength is categorized as weak or none (r < 0.3), weak (0.3 ≤ r < 0.5), moderate (0.5 ≤ r < 0.8), and strong (r ≥ 0.8), with red indicating positive and blue indicating negative correlations.

The optimal soft threshold for WGCNA was determined as 6 based on scale-free topology and network connectivity (**Figure 3A**). WGCNA on the top 5,000 significant DEGs identified OSCC co-expression modules, with a minimum soft threshold of 6 (index=0.96) for network construction. These DEGs were clustered into 9 modules (**Figure 3B**). Module gene expression analysis revealed correlations with IAPS in the OSCC cohort (**Figure 3C**). The top 5,000 high-variance genes were clustered, and their relationships with merged modules were visualized (**Figure 3D**). Four modules (Black, Blue, Red, Yellow; |r| > 0.3) were selected for further analysis. Venn diagrams (**Figures 3E–H**) intersected the 53 IR&PRDEGs with these modules, identifying 12 module genes, including: *CTSG, COL1A1, MMP9, NR4A1, SERPINH1, PTX3, CRTAC1, TCEA3, DEPTOR, SPP1, PCSK9*, and *HKDC1*.

### 3.7 Screening of prognostic genes in oral squamous cell carcinoma

To build an OSCC prognostic risk model, module genes from TCGA-HNSC OSCC samples were analyzed using univariate Cox regression (see **Supplementary Figure 4A**). Genes with P<0.1 were selected for LASSO regression, visualized in model and variable trajectory diagrams (**Supplementary Figure 4B, C**). The LASSO model identified four prognostic genes: CTSG, HKDC1, PTX3, and SPP1, with their multivariate regression analysis shown in a forest plot (**Supplementary Figure 4D**).

### 3.8 Prognostic analysis of Oral squamous cell carcinoma and internal validation of the model

**Figure 4:**
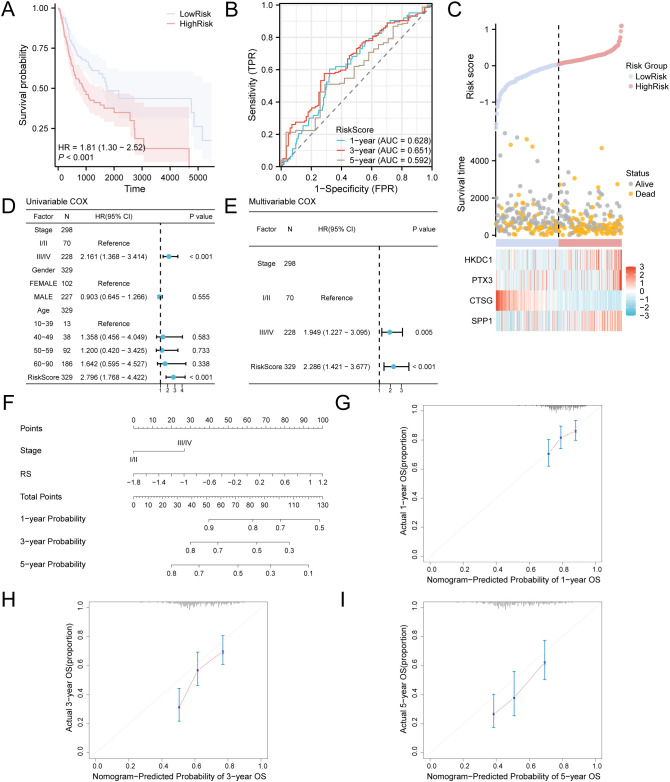
Prognostica nalysis and model internal validation. **(A)** Kaplan-Meier survival analysis comparing high and low-risk score groups derived from LASSO for overall survival (OS) in oral squamous cell carcinoma (OSCC). **(B)** Time-dependent receiver operating characteristic (ROC) curves for OSCC samples from the TCGA-HNSC dataset. **(C)** Risk factor plot illustrating the relationship between risk scores, OS, and prognostic genes. D-E. Univariate Cox regression analysis **(D)** and multivariate Cox regression analysis forest plot **(E)** for the prognostic risk model in OSCC. **(F)** Nomograms predicting 1-, 3-, and 5-year survival probabilities based on the OSCC prognostic risk model. **(G–I)** Calibration curves for the prognostic risk model at 1 year **(G)**, 3 years **(H)**, and 5 years **(I)** in OSCC.


Kaplan-Meier analysis (**Figure 4A**) split OSCC samples into high- and low-risk groups by median overall survival, revealing significant survival differences (P<0.001). Time-dependent ROC curves (**Figure 4B**) showed varying accuracy of the OSCC risk score at 1, 3, and 5 years, peaking at 3 years. A risk factor triangle plot (**Figure 4C**) was generated using the risk score, overall survival, and prognostic gene expression. The risk score calculation method is detailed below.

RiskScore = CTSG*(−0.3850)+HKDC1*0.0707+PTX3*0.1230+SPP1*0.0408

A prognostic risk model including risk score, age, gender, and clinical stage was analyzed using univariate Cox regression (**Figure 4D**), confirming that risk score and clinical stage significantly correlated with OS (P<0.1). Multivariate Cox regression (**Figure 4E**) identified risk score and stage as independent prognostic factors (P<0.05). See **Table 1** for details. The nomogram of the prognostic risk model (**Figure 4F**) demonstrated that the risk score is more effective than clinical stage in predicting 1-, 3-, and 5-year survival in the OSCC model. Calibration curves (**Figures 4G–I**) showed that the 1-year survival prediction closely matched the ideal, while the 3- and 5-year predictions were overestimated.

**Table 1:** Results of univariable and multivariable cox analysis for TCGA-HNSC OSCC.

Characteristics	Total(N)	Univariate analysis		Multivariate analysis	
		Hazard ratio (95% CI)	P value	Hazard ratio (95% CI)	P value
Stage	298				
I/II	70	Reference		Reference	
III/IV	228	2.161 (1.368 - 3.414)	< 0.001	1.949 (1.227 - 3.095)	0.005
Gender	329				
FEMALE	102	Reference			
MALE	227	0.903 (0.645 - 1.266)	0.555		
Age	329				
10-39	13	Reference			
40-49	38	1.358 (0.456 - 4.049)	0.583		
50-59	92	1.200 (0.420 - 3.425)	0.733		
60-90	186	1.642 (0.595 - 4.527)	0.338		
RiskScore	329	2.796 (1.768 - 4.422)	< 0.001	2.286 (1.421 - 3.677)	< 0.001

TCGA, The Cancer Genome Atlas;HNSC, Head and Neck Squamous Cell Carcinoma;OSCC, Oral Squamous Cell Carcinoma.

### 3.9 Prognostic analysis of oral squamous cell carcinoma and external validation of the model

**Figure 5:**
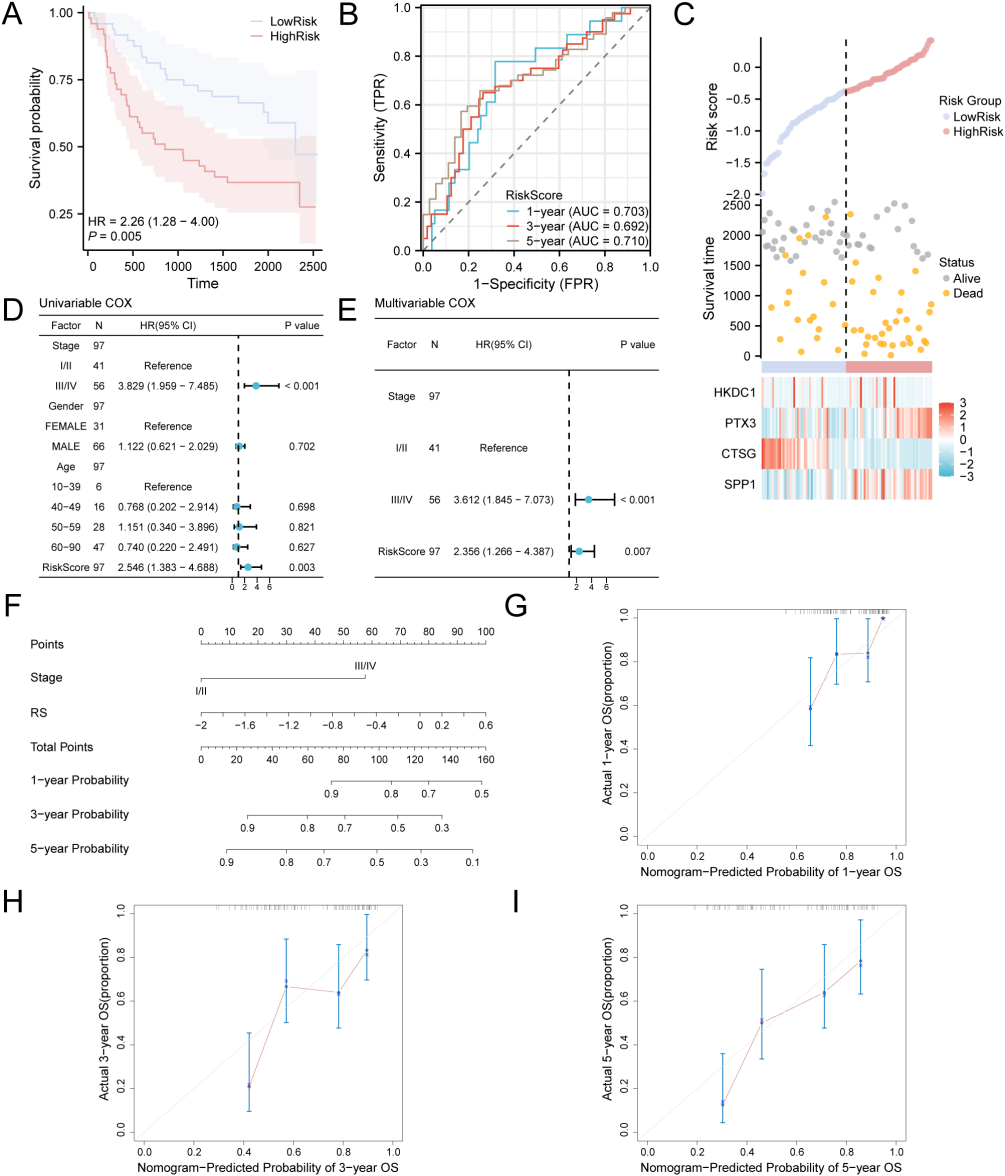
Prognostic analysis and model external validation. **(A)** Kaplan-Meier survival curve comparing high and low-risk score groups as determined by LASSO analysis and overall survival (OS) in oral squamous cell carcinoma (OSCC). **(B)** Time-dependent receiver operating characteristic (ROC) curve for OSCC samples derived from the GSE41613 dataset. **(C)** Risk factor plot illustrating the relationship between risk score, OS, and prognostic genes. **(D, E)** Univariate **(D)** and multivariate **(E)** Cox regression analyses presented as a forest plot for the prognostic risk model in OSCC. **(F)** Nomograms predicting 1-, 3-, and 5-year survival probabilities based on the OSCC prognostic risk model. **(G–I)** Calibration curves for the prognostic risk model of OSCC at 1 year **(G)**, 3 years **(H)**, and 5 years **(I)**.


Kaplan-Meier analysis of GSE41613 OSCC samples, grouped by median risk score, revealed significant survival differences between high- and low-risk groups (P<0.05; **Figure 5A**). Time-dependent ROC curves (**Figure 5B**) showed the risk score had moderate accuracy at 1- and 5-year intervals (0.7<AUC<0.9, highest at 5 years) but low accuracy at 3 years (0.5<AUC<0.9). **Figure 5C** illustrates a risk factor triad comprising the risk score, OS, and prognostic gene expression. The risk score calculation method is detailed below.

RiskScore = CTSG*(−0.3850)+HKDC1*0.0707+PTX3*0.1230+SPP1*0.0408

A prognostic risk model, including risk score, age, gender, and clinical stage, was analyzed using univariate Cox regression (**Figure 5D**), revealing that risk score and clinical stage significantly affect overall survival (P<0.1). Multivariate Cox regression (**Figure 5E**) confirmed them as independent prognostic factors (P<0.05). The prognostic risk model’s nomogram (**Figure 5F**) highlights the greater predictive value of the risk score over clinical stage for 1-, 3-, and 5-year survival in the OSCC model. Calibration curves (**Figures 5G–I**) show the model’s predictions on the horizontal axis and actual outcomes on the vertical axis, with the model performing best for 1-year survival predictions.

### 3.10 Differential analysis of prognostic gene expression

**Figure 6:**
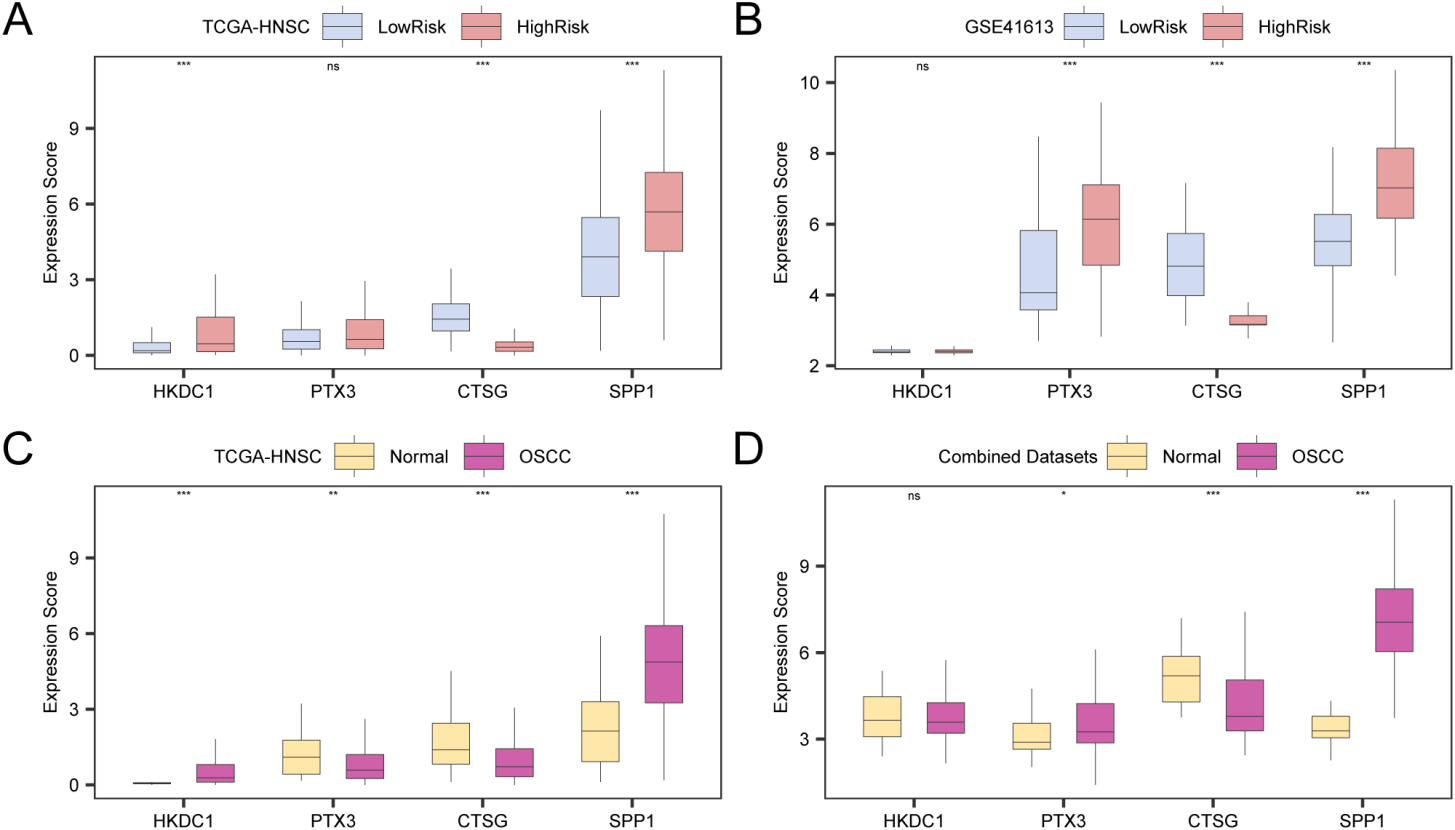
Differential expression of prognostic genes. **(A)** Analysis of prognostic gene variability between high-risk and low-risk groups in OSCC samples from the TCGA-HNSC dataset. **(B)** Evaluation of prognostic gene differences between high-risk and low-risk groups in OSCC samples from the GSE41613 dataset. **(C)** Comparative analysis of prognostic genes between OSCC and control (normal) groups in the TCGA-HNSC dataset. **(D)** Comparative visualization of prognostic genes between OSCC and control (normal) groups across the combined GEO dataset of GSE23558 and GSE25099. Light blue represents the low-risk group, pink indicates the high-risk group, yellow denotes the control (Normal) group, and purple signifies the OSCC group. ns, P ≥ 0.05; *P < 0.05; **P < 0.01; *** P < 0.001.

To examine expression differences of prognostic genes in high- and low-risk OSCC samples, samples from TCGA-HNSC and GSE41613 were divided by median risk score. Group comparison plots (**Figures 6A, B**) illustrate the expression of four prognostic genes in these groups from the TCGA-HNSC dataset. Results indicated significant expression differences in prognostic genes between high- and low-risk OSCC samples in TCGA-HNSC (P<0.05; **Figure 6A**): *HKDC1, CTSG*, and *SPP1*. In GSE41613, *PTX3*, *CTSG*, and *SPP1* also showed significant differences between high- and low-risk OSCC samples (P<0.05; **Figure 6B**). Comparisons of OSCC and control groups in TCGA-HNSC and GEO datasets revealed significant differences for *HKDC1*, *PTX3*, *CTSG*, and *SPP1* in TCGA-HNSC (P<0.05; **Figure 6C**), and *PTX3*, *CTSG*, and *SPP1* in the combined GEO dataset of GSE23558 and GSE25099 (P<0.05; **Figure 6D**).

### 3.11 Gene set enrichment analysis

TCGA-HNSC OSCC samples were divided into high-risk and low-risk groups. DESeq2 (v1.42.0) identified 195 DEGs with |logFC| > 2 and adj. P < 0.05: 94 up-regulated and 101 down-regulated, shown in a volcano plot (**Supplementary Figure 5A**) and heatmap (**Supplementary Figure 5B**). GSEA linked gene expression levels to biological processes, cellular components, and molecular functions (**Supplementary Figure 5C**, **Supplementary Table 9**), revealing significant enrichment in pathways like anti-inflammatory response to leishmania infection (**Supplementary Figure 5D**), IL12 pathway (**Supplementary Figure 5E**), inflammatory response (**Supplementary Figure 5F**), and inflammatory bowel disease signaling (**Supplementary Figure 5G**), among others.

### 3.12 Construction of protein-protein interaction network and mRNA-miRNA regulatory network

The GeneMANIA website predicted the interaction network of four model genes and their functionally similar genes, with colored lines indicating co-expression and shared features (**Supplementary Figure 6A**). This included 4 prognostic genes and 20 similar proteins, detailed in **Supplementary Table 10**. We then used the StarBase database to find miRNAs associated with the prognostic genes and visualized the mRNA-miRNA regulatory network using Cytoscape (**Supplementary Figure 6B**), involving 2 prognostic genes and 45 miRNAs, detailed in **Supplementary Table 11**.

### 3.13 Immune infiltration analysis

The ssGSEA algorithm assessed immune infiltration in 28 cell types using TCGA-HNSC OSCC sample data. A comparison plot (**Supplementary Figure 7A**) identified significant infiltration differences in 27 cell types (P < 0.05). Correlation heatmaps (**Supplementary Figures 7B, C**) showed strong positive correlations among these cells: Regulatory T cells and MDSCs were highest in the low-risk group (R = 0.919, P < 0.05), and Type 1 T helper cells and MDSCs in the high-risk group (R = 0.909, P < 0.05). Correlation bubble plots (**Supplementary Figures 7D, E**) linked prognostic genes and immune infiltration: in the low-risk group, gene *CTSG* and Mast cells had the strongest correlation (r=0.707, P < 0.05), while in the high-risk group, *CTSG* and Mast cells were most correlated (R = 0.479, P < 0.05).

Using the ssGSEA algorithm on the TCGA-HNSC expression matrix, we evaluated immune cell infiltration for 28 types. A comparison plot (**Supplementary Figure 8A**) identified 13 immune cell types with significant infiltration differences between OSCC and controls (P < 0.05), including Activated CD4 T cells and Regulatory T cells. A correlation heatmap (**Supplementary Figure 8B**) showed strong positive correlations among these 13 types, especially between Central memory CD4 T cells and Regulatory T cells (R = 0.847, P < 0.05). A correlation bubble plot (**Supplementary Figure 8C**) highlighted links between prognostic genes and immune cell infiltration, with the strongest being between gene *CTSG* and Mast cells (R = 0.747, P < 0.05).

### 3.14 TIDE, MSI and TMB analysis

**Figure 7:**
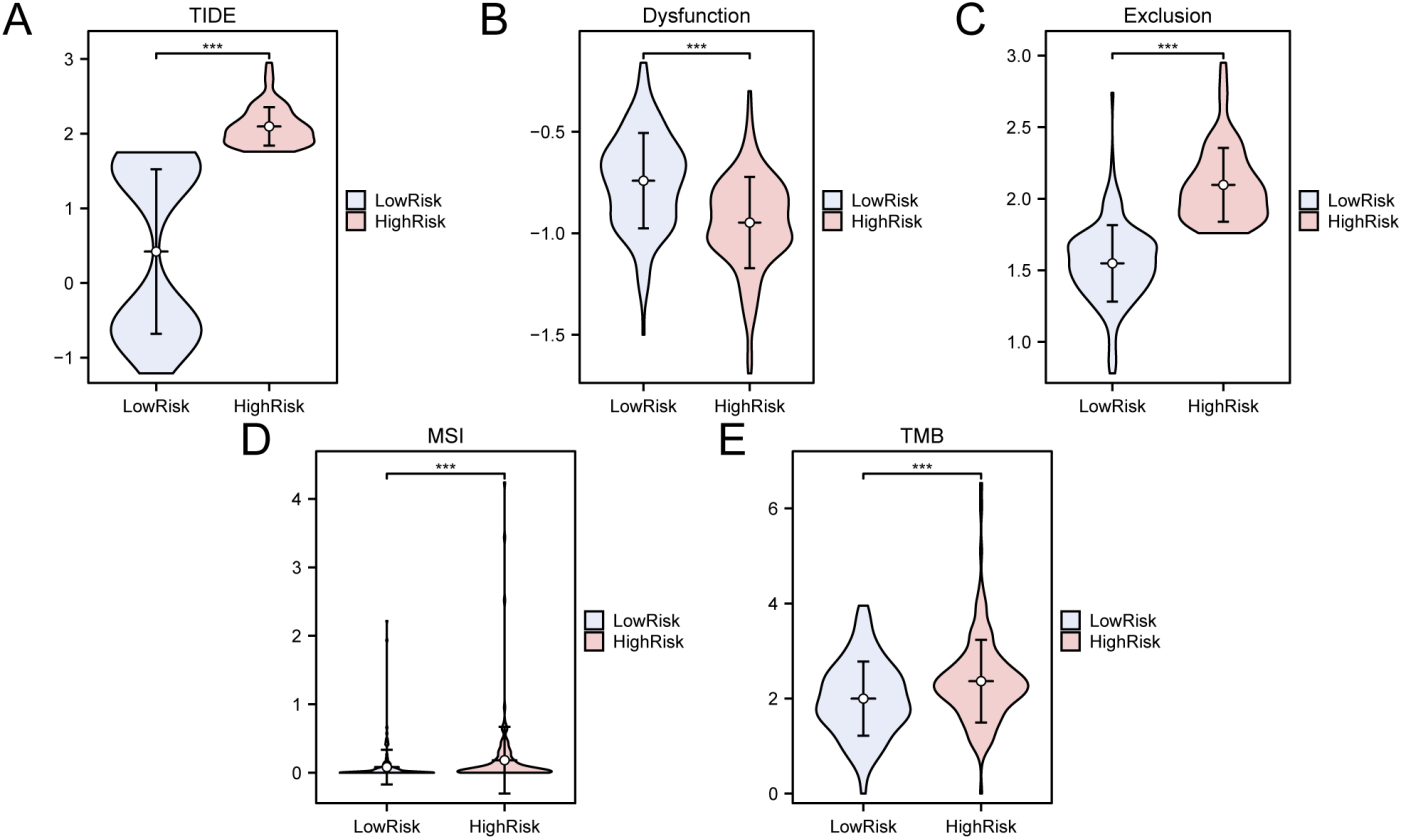
TIDE, MSI and TMB analysis. The results of the TIDE immunotherapy scores for low-risk and high-risk groups of oral squamous cell carcinoma (OSCC) samples from the TCGA-HNSC dataset are presented in panels **(A–E)**. Specifically, panel **(A)** illustrates the comparative analysis of TIDE scores, while panels B focus on T cell immune dysfunction, **(C)** focus on T cell dysfunction, and **(D)** focus on MSI score, and panel **(E)** addresses TMB score. ***P< 0.001.

We evaluated the immunotherapy sensitivity of TCGA-HNSC OSCC samples using the TIDE algorithm, with results shown in group comparison plots. The TIDE scores revealed significant differences between low-risk and high-risk groups (P<0.001; **Figure 7A**), with lower scores in the low-risk group, suggesting better immunotherapy response. T cell dysfunction was higher in the low-risk group (P<0.001; **Figure 7B**), while T cell exclusion was lower compared to the high-risk group (P<0.001; **Figure 7C**).

We compared microsatellite instability (MSI) and tumor mutation burden (TMB) scores between low-risk and high-risk OSCC samples in the TCGA-HNSC dataset. The MSI scores (**Figure 7D**) showed significant differences (P<0.001), with lower scores in the low-risk group. Similarly, TMB scores (**Figure 7E**) also revealed significant differences (P<0.001), with the low-risk group having lower scores than the high-risk group.

### 3.15 Verification of prognostic gene expression in oral cancer cells by wet experiments

**Figure 8:**
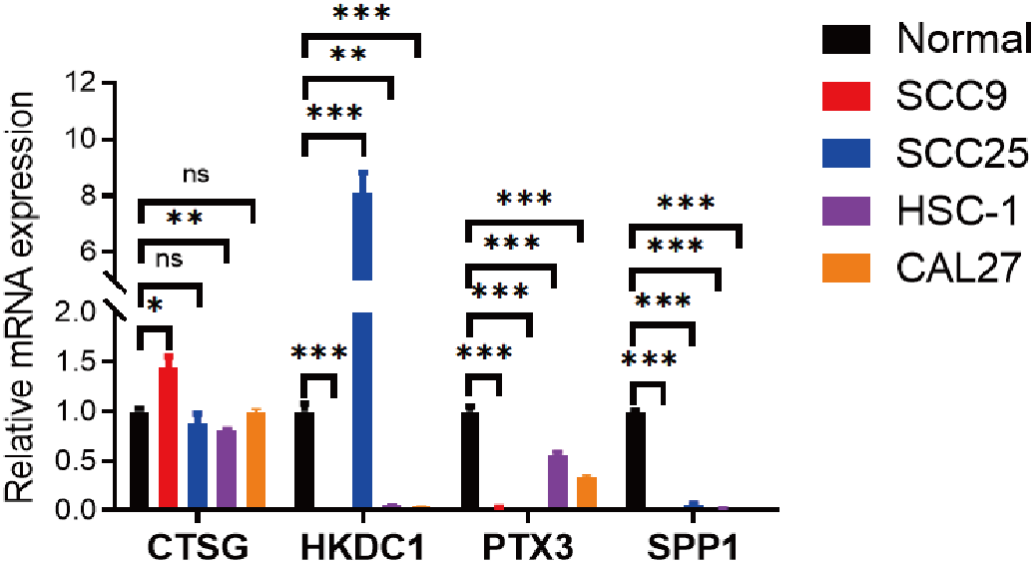
Bar graph illustrating the relative mRNA expression levels of four genes (*CTSG, HKDC1, PTX3, SPP*1) across different cell lines, including normal, SCC9, SCC25, HSC - 1, and CAL27. ns, P ≥ 0.05; *P < 0.05; **P < 0.01; ***P < 0.001.

The mRNA expression levels of *CTSG*, *HKDC1*, *PTX3*, and *SPP1* were assessed in Normal, SCC9, SCC25, HSC-1, and CAL27 cell lines (**Figure 8**). *CTSG* was significantly higher in SCC9 and lower in HSC-1 compared to Normal, with no significant changes in other lines. *HKDC1* was upregulated in all cancer lines except SCC25. *PTX3* and *SPP1* were significantly downregulated in most cancer lines compared to Normal (P<0.001). These results reveal distinct gene expression patterns in oral cancer cell lines.

## 4 Discussion

Oral squamous cell carcinoma significantly diminishes patients’ quality of life by affecting essential functions such as chewing, swallowing, speech, and facial appearance, while also imposing substantial economic burdens on healthcare systems. Despite advancements in surgical, radiotherapeutic, and chemotherapeutic interventions, the prognosis for OSCC remains unfavorable (22, 23). The progression of OSCC is characterized by intricate biological processes, with inflammation—shaping the tumor microenvironment (24, 25) and promoting progression—and pyroptosis—a form of programmed cell death closely associated with tumorigenesis—playing pivotal roles. Although bioinformatics has facilitated tumor gene research, studies on OSCC predominantly concentrate on individual genes or small gene groups. There is a notable deficiency in systematic investigations into the interplay between inflammation and pyroptosis, their combined effects on OSCC progression and prognosis, and the precise identification of key genes for prognostic biomarkers, therapeutic targets, and clinically relevant prognostic models, underscoring critical research needs.

This study utilized the GeneCards and MSigDB databases to identify and integrate inflammation-related genes with pyroptosis-related genes, resulting in the identification of 513 overlapping genes, referred to as IR&PRGs. From these, 53 differentially expressed IR&PRGs were further analyzed within the TCGA-HNSC cohort. Functional enrichment analyses, including GO and KEGG, revealed significant enrichment of these genes in biological processes and pathways such as the “cytokine-mediated signaling pathway” and “neutrophil extracellular trap formation”. Notably, the enrichment of systemic lupus erythematosus-related pathways merely suggests the potential existence of shared immune-inflammatory signaling regulatory mechanisms, rather than indicating a direct correlation with this disease.These findings corroborate previous studies, such as Gao et al. (26), who demonstrated that pyroptosis-associated genes influence inflammatory responses through the NOD-like receptor signaling pathway, and Zhai et al.,Garley et al. and Siquara da Rocha et al. (27–29), who showed that neutrophil extracellular traps (NETs) facilitate OSCC metastasis. The primary innovation of this study is its systematic integration of inflammatory and pyroptosis pathways, providing evidence that IR&PRDEGs contribute to OSCC progression by orchestrating a sequential cascade involving “inflammatory amplification–pyroptosis activation–NET formation.” This offers a more comprehensive mechanistic understanding of OSCC pathogenesis. Subsequent GSEA analysis demonstrated that genes in the high-risk cohort were significantly enriched in pathways such as “oxidative phosphorylation” and “drug metabolism-related pathways.” In contrast, the low-risk cohort exhibited enrichment in the “B cell receptor signaling pathway” and “T cell receptor signaling pathway.” These observations are consistent with the findings of Zeng et al. And Liu et al. (30, 31), whose pyroptosis-related prognostic model for OSCC similarly suggested increased metabolic activity and diminished immune function in high-risk patients. Importantly, our study is the first to identify that *HKDC1*,a differentially expressed gene related to inflammation and pyroptosis, may participate in the regulation of glycolytic metabolism,and thus be associated with risk stratification of OSCC. Despite its high expression in OSCC tissues, *HKDC1* expression is reduced in high-risk cases. This indicates that *HKDC1* may function as a potential “metabolic–inflammatory” switch, promoting glycolytic metabolism to support tumor proliferation in early stages, while potentially facilitating immune evasion through the suppression of inflammatory responses in advanced disease. Currently, the results only support an association between *HKDC1* and risk stratification as well as related biological characteristics, and its specific dual functions require further mechanistic experiments for verification. This potential dual role may provides a novel theoretical basis for metabolism-targeted interventions in OSCC.

Phenotypic correlation analysis in the present study revealed that tumor mutational burden (TMB) and microsatellite instability (MSI) scores were markedly higher in patients of the high-risk group than in those of the low-risk group (P < 0.001). Of note, tumor immune dysfunction and exclusion (TIDE) scores were also elevated in the high-risk group (P < 0.001). This evident contradiction contradicts the mainstream view that high TMB generally indicates enhanced sensitivity to immunotherapy. In contrast, our research results are consistent with the recent findings of Liu et al. (29), who reported that high TMB in oral squamous cell carcinoma (OSCC) is often correlated with upregulated programmed death-ligand 1 (PD-L1) expression and increased regulatory T cell infiltration. The coexistence of high TMB and high TIDE scores may suggest more complex immune evasion characteristics in the high-risk group. The coexistence of high TMB and high TIDE scores may suggest more complex immune evasion characteristics in the high-risk group, but this interpretation requires further validation.

The findings of this study significantly elucidate the fundamental direction for the precise diagnosis and treatment of OSCC, offering comprehensive support for clinical practice. The current TNM staging system does not adequately capture molecular heterogeneity. In contrast, the 4-gene model developed in this study, incorporating *CTSG, HKDC1, PTX3*, and *SPP1*, quantifies risk, effectively differentiates between high-risk and low-risk patients, and serves as an independent prognostic factor (P<0.05). This aligns with the “molecular + clinical” evaluation strategy proposed by Qi et al. (32), addressing the limitations of the traditional staging system. Regarding immunotherapy, the low-risk group demonstrates higher infiltration of activated CD8+ T cells and a lower TIDE score, corroborating the findings of Gao et al. (26) that “the low-score group has a high response rate to PD-1 inhibitors.” Additionally, this study is the first to identify that *CTSG* exhibits the strongest positive correlation with mast cell infiltration (r=0.707, P<0.05). In conjunction with the findings of Siquara da Rocha et al. (29), which demonstrate that mast cells are capable of releasing NET-like structures, our study suggests that *CTSG* may serve as a viable target for combined immunotherapy. This presents a novel strategy for enhancing the sensitivity of OSCC to immunotherapeutic interventions. In the context of developing therapeutic targets, *PTX3* is implicated in promoting cancer progression, *SPP1* is involved in the renewal of cancer stem cells, and *HKDC1* exhibits characteristics of “bidirectional regulation.” When considered alongside the concept of metabolic intervention for OSCC, these factors collectively offer potential candidate targets for the precise treatment of OSCC.

We performed cellular experiments to examine the expression of four prognostic genes (*CTSG*, *HKDC1, PTX3*, and *SPP1*) in OSCC cell lines, but these provided only limited validation for the bioinformatics predictions. The results showed considerable variation across cell lines and did not fully support the bioinformatics findings.The mismatch between qRT-PCR data and bioinformatics analysis likely reflects differences in sample composition: bulk tissues contain tumor cells, immune cells, and stromal cells, whereas cell lines represent only a single tumor cell population grown *in vitro*. OSCC cell lines also differ in their molecular characteristics, epigenetic states, and post-transcriptional regulation, which can produce divergent expression patterns.Without large-scale experimental validation, the reliability and clinical utility of these biomarkers remain uncertain. Future work should combine multi-omics approaches with *in vivo* and *in vitro* experiments, along with validation in clinical tissue samples, to better establish the robustness and clinical relevance of these results.

A notable limitation of this research is its reliance on bioinformatics methodologies, which are inherently contingent upon the quality and availability of existing genomic data. Although this study has preliminarily identified differential expression of inflammation- and pyroptosis-related genes, these findings may not fully encapsulate the complexity of OSCC biology due to potential confounding variables such as tumor heterogeneity, variations in the microenvironment, and patient-specific factors. It should be noted that the current study’s interpretation of the mechanisms related to high TMB and immune escape is still mainly based on the results of TIDE, MSI/TMB, and immune infiltration. In the future, further verification is needed by combining immune checkpoint expression, immunosuppressive cell infiltration, and real-world immunotherapy cohorts.

## 5 Conclusion

This study develops a prognostic risk model for OSCC predicated on the interplay of genes linked to inflammation and pyroptosis. The research identifies *CTSG,HKDC1,PTX3* and *SPP1* as independent prognostic indicators and elucidates significant correlations between risk scores and characteristics of the tumor microenvironment, metabolic activity, and therapeutic sensitivity. This model may provide a reference for OSCC prognosis predictions and offers novel insights for future studies to further explore its potential relevance to treatment stratification. The findings align closely with existing literature while addressing a critical knowledge gap concerning the synergistic regulatory mechanisms between inflammation and pyroptosis. These outcomes possess substantial theoretical significance and potential for clinical translation.

## Data Availability

The original contributions presented in the study are included in the article/**Supplementary Material**. Further inquiries can be directed to the corresponding author.
