# Supplementary figures and images for: Identification and validation of a prognostic signature comprising inflammation and pyroptosis-related genes in oral squamous cell carcinoma
Source: Front Immunol. 2026 Jul 7;17:1721849. doi: 10.3389/fimmu.2026.1721849 (PMC13384851; doi:10.3389/fimmu.2026.1721849)

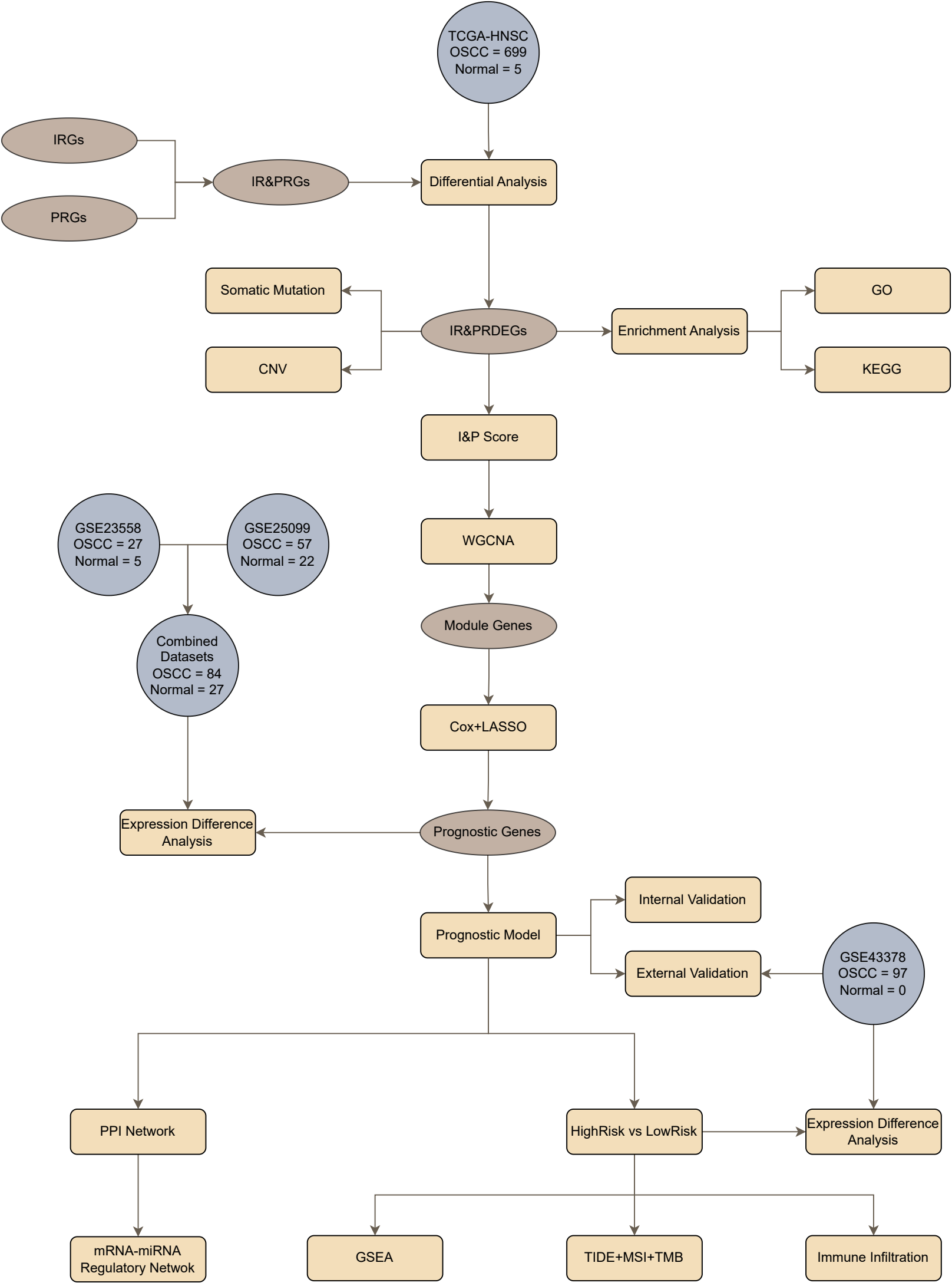

Supplement: Supplementary file 6 [file DataSheet6.pdf]

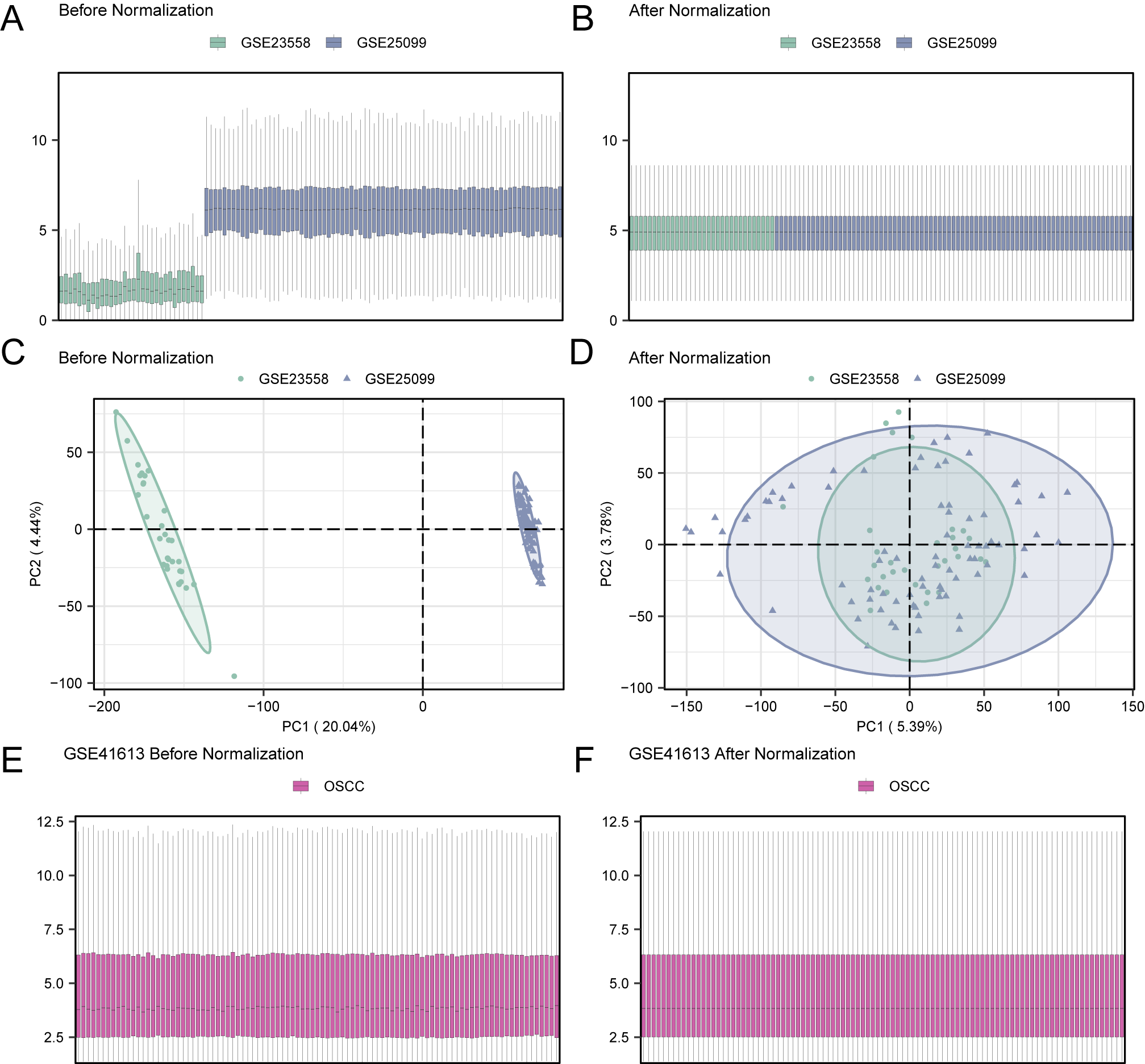

Supplement: Supplementary file 7 [file Image1.tif]

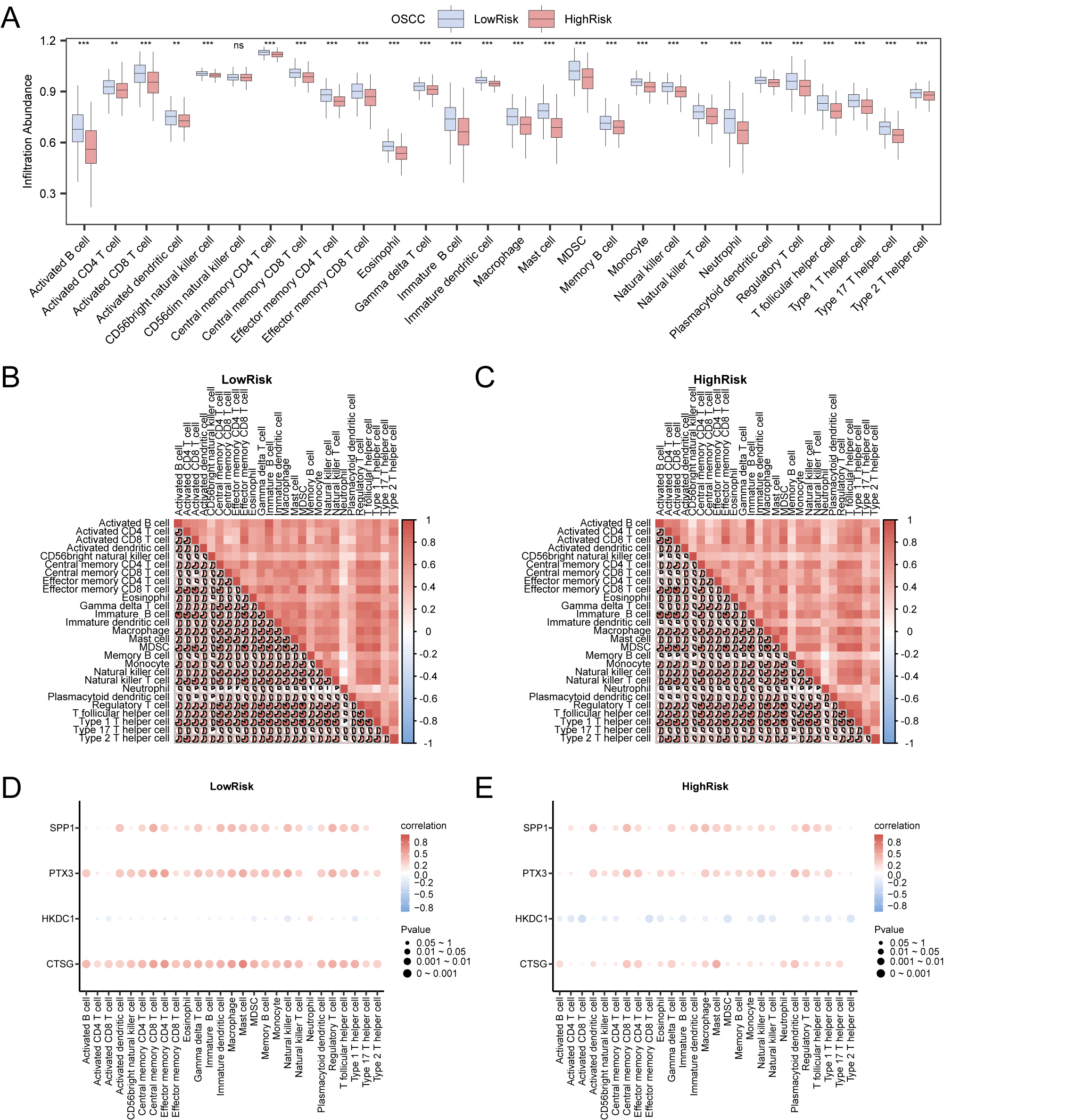

Supplement: Supplementary file 8 [file Image2.tif]

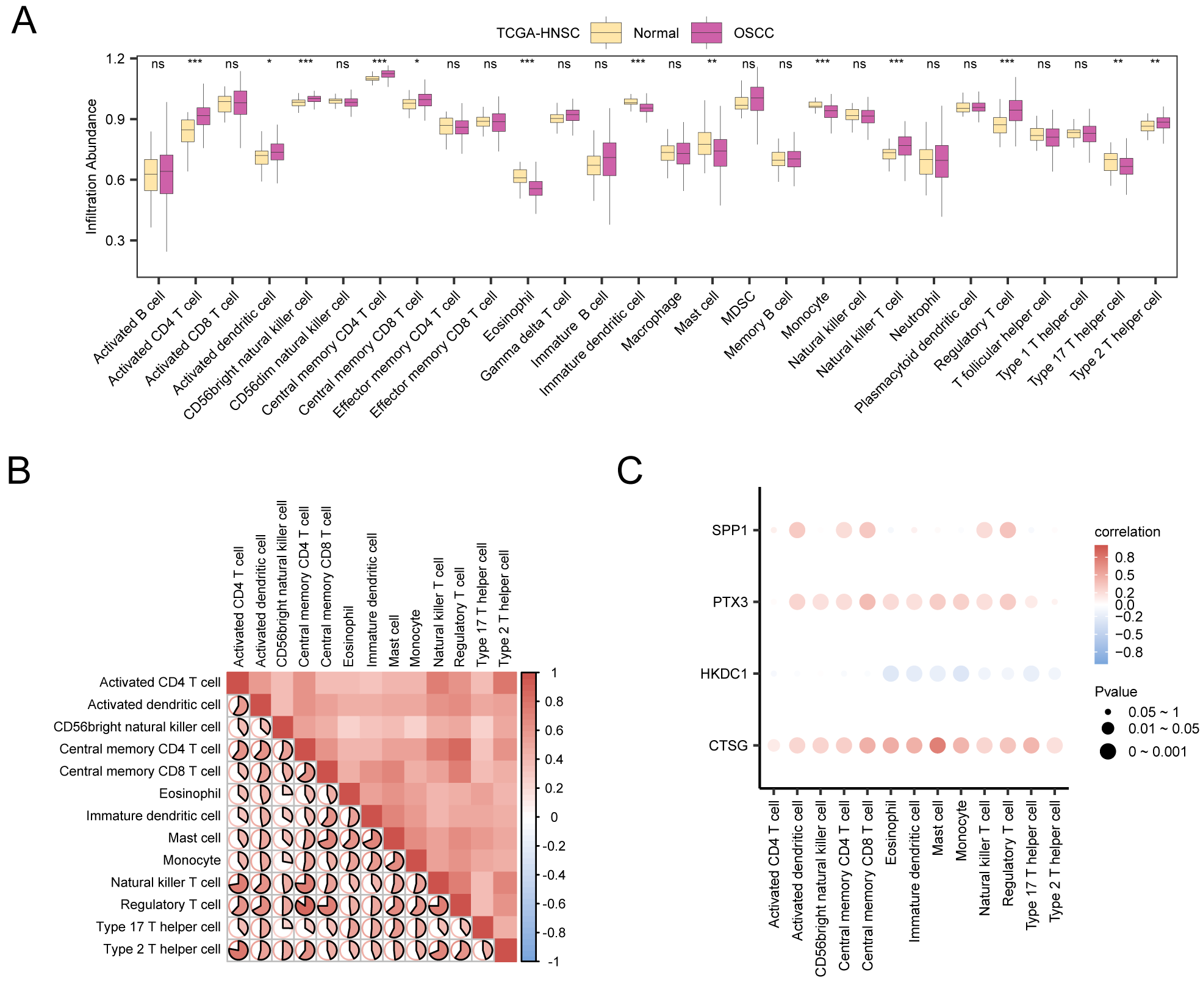

Supplement: Supplementary file 9 [file Image3.tif]

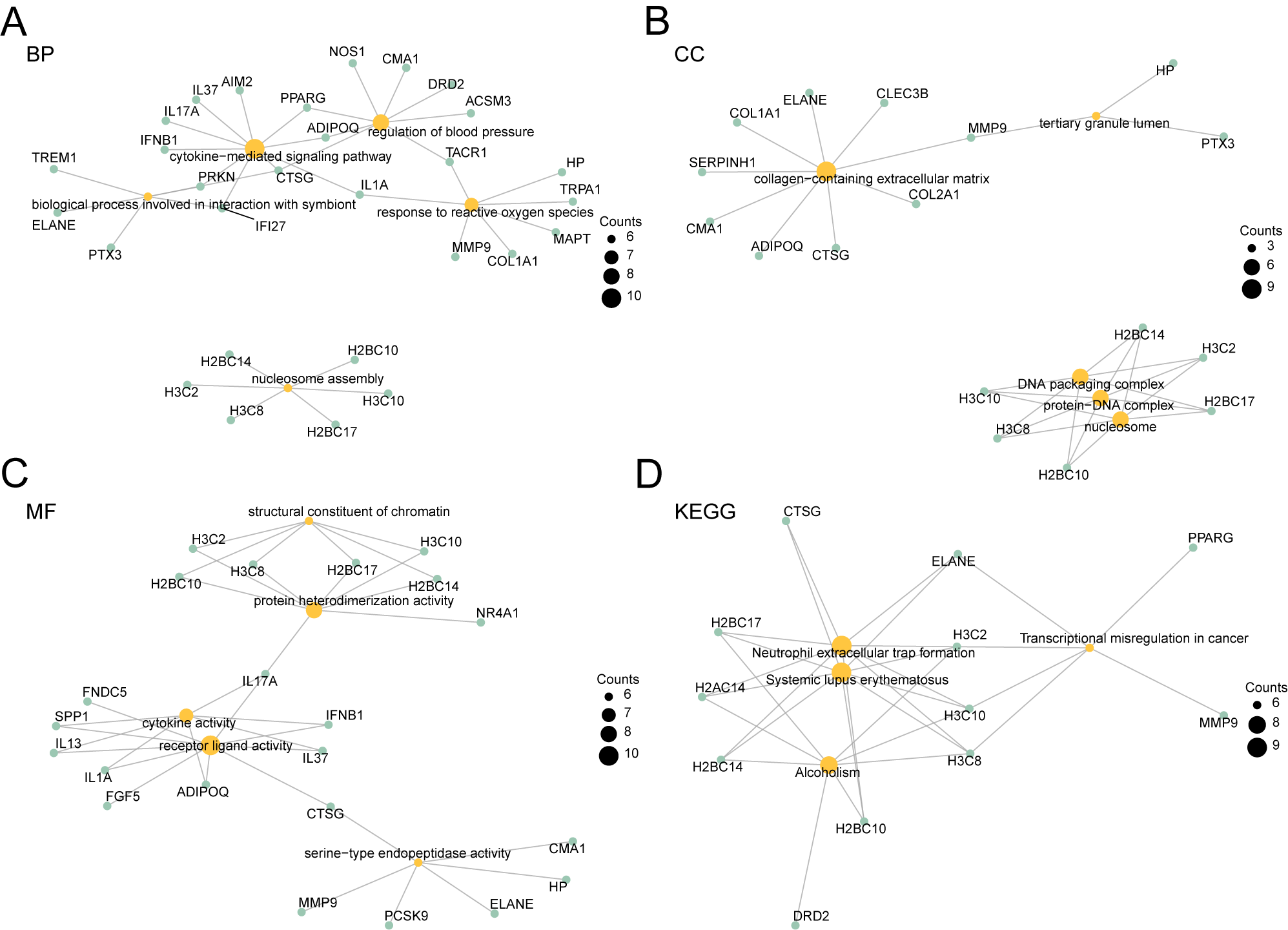

Supplement: Supplementary file 10 [file Image4.tif]

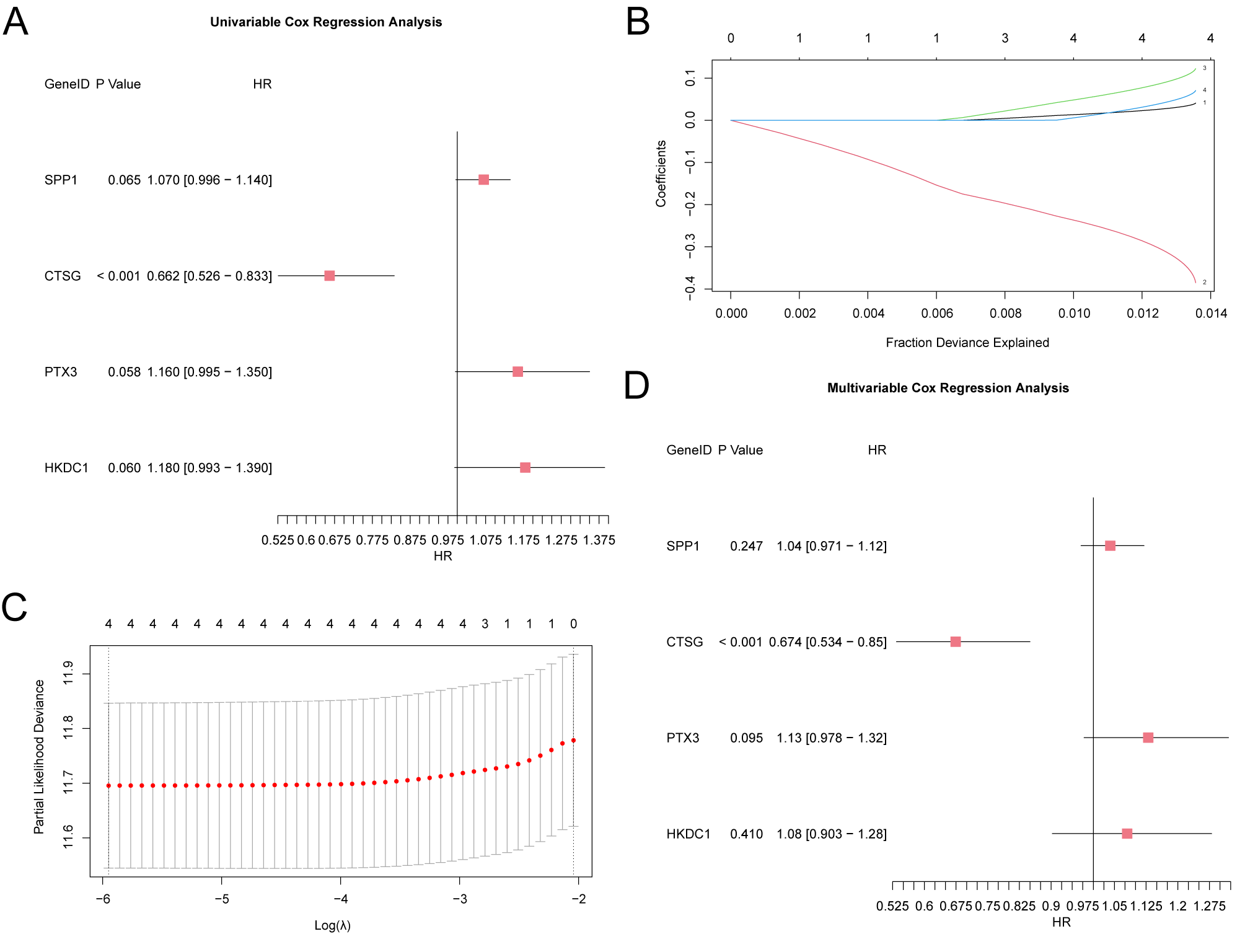

Supplement: Supplementary file 11 [file Image5.tif]

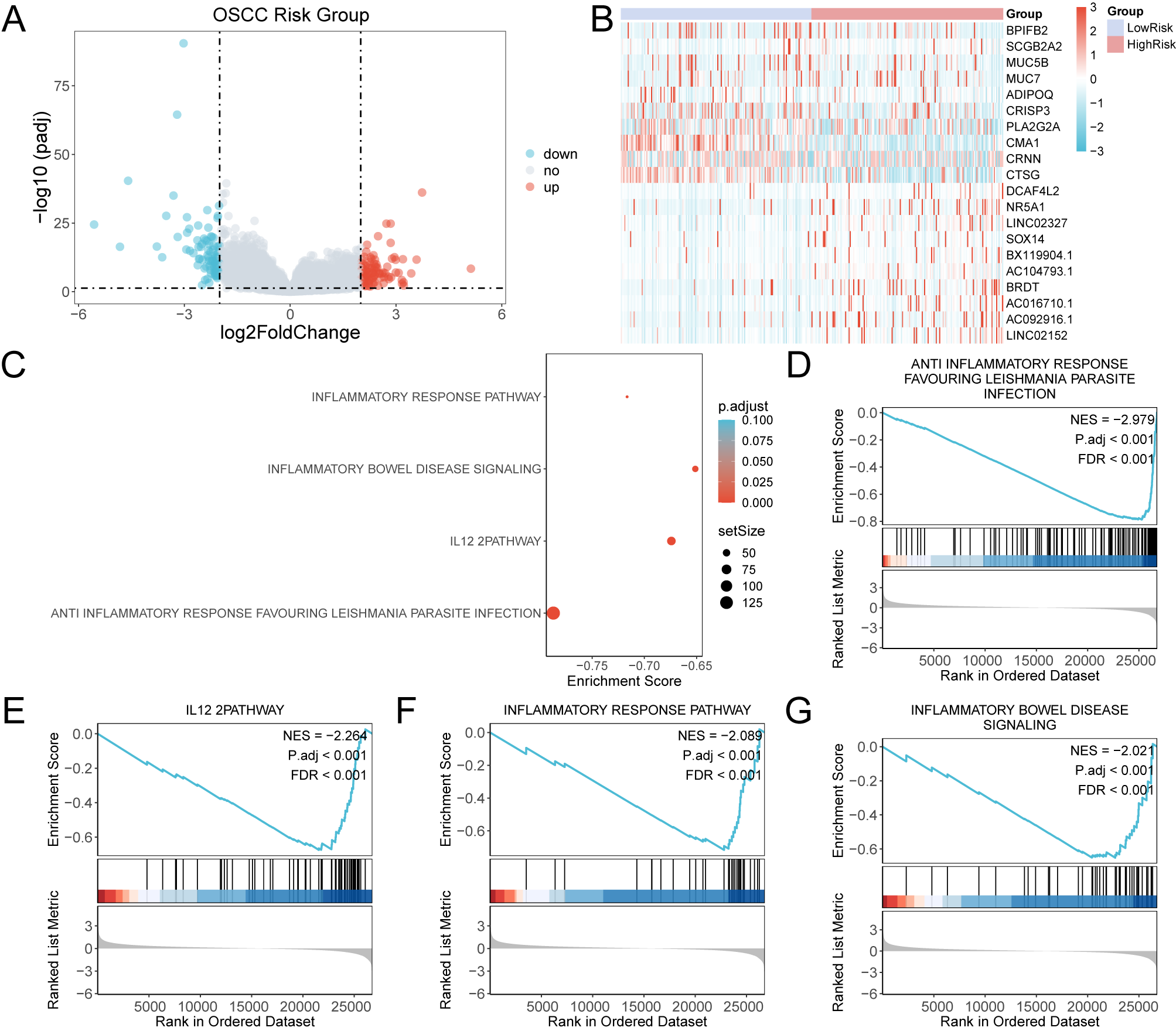

Supplement: Supplementary file 12 [file Image6.tif]

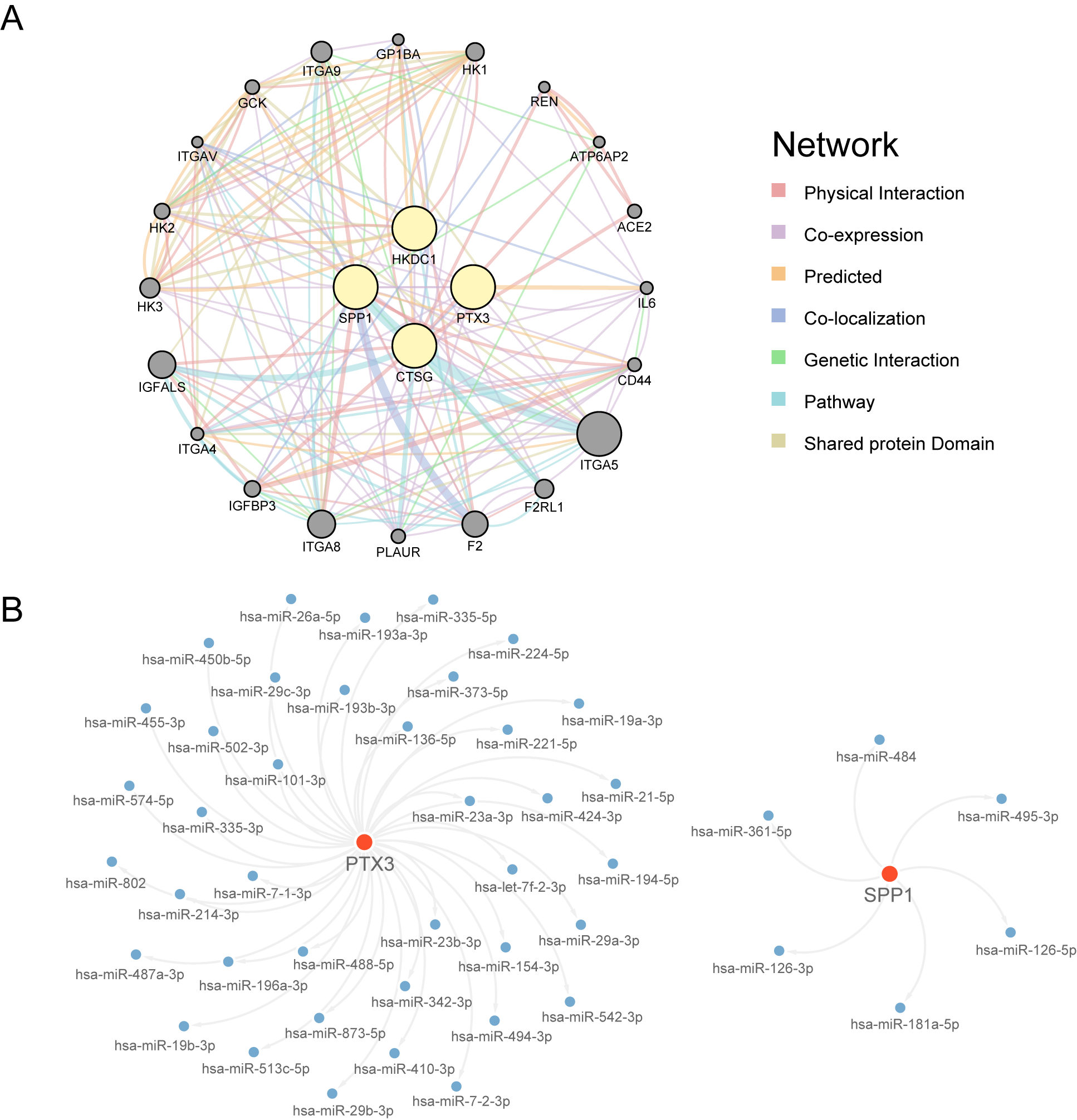

Supplement: Supplementary file 13 [file Image7.tif]
