# Supplementary material for: Identification and validation of a prognostic signature comprising inflammation and pyroptosis-related genes in oral squamous cell carcinoma
Source: Front Immunol. 2026 Jul 7;17:1721849. doi: 10.3389/fimmu.2026.1721849 (PMC13384851; doi:10.3389/fimmu.2026.1721849)
Supplement: Supplementary file 14 [file Table1.docx]

**Table S1 Baseline Table with TCGA-HNSC OSCC Patients Characteristics**

| Characteristics | Overall |
| --- | --- |
| n | 329 |
| Stage (%) |  |
| I/II | 70 (23.5) |
| III/IV | 228 (76.5) |
| Gender (%) |  |
| FEMALE | 102 (31.0) |
| MALE | 227 (69.0) |
| Age (%) |  |
| 10-39 | 13 (4.0) |
| 40-49 | 38 (11.6) |
| 50-59 | 92 (28.0) |
| 60-90 | 186 (56.5) |

TCGA，The Cancer Genome Atlas；HNSC，Head and Neck Squamous Cell Carcinoma；OSCC，Oral Squamous Cell Carcinoma。
