# Supplementary material for: Identification and validation of a prognostic signature comprising inflammation and pyroptosis-related genes in oral squamous cell carcinoma
Source: Front Immunol. 2026 Jul 7;17:1721849. doi: 10.3389/fimmu.2026.1721849 (PMC13384851; doi:10.3389/fimmu.2026.1721849)
Supplement: Supplementary file 15 [file Table2.docx]

## Table S2GEO Microarray Chip Information

|  | GSE23558 | GSE25099 | GSE41613 |
| --- | --- | --- | --- |
| Platform | GPL6480 | GPL5175 | GPL570 |
| Type | Array | Array | Array |
| Species | Homo sapiens | Homo sapiens | Homo sapiens |
| Tissue | Oral | Oral | Oral |
| Samples in OSCC group | 27 | 57 | 97 |
| Samples in Normal group | 5 | 22 | 0 |
| Reference | PMID: 22072328，PMID: 28433800，PMID: 37245006 | PMID: 21853135 | PMID: 23319825，PMID: 35464860 |

GEO，Gene Expression Omnibus；OSCC，Oral Squamous Cell Carcinoma。
