# Supplementary material for: Identification and validation of a prognostic signature comprising inflammation and pyroptosis-related genes in oral squamous cell carcinoma
Source: Front Immunol. 2026 Jul 7;17:1721849. doi: 10.3389/fimmu.2026.1721849 (PMC13384851; doi:10.3389/fimmu.2026.1721849)
Supplement: Supplementary file 16 [file Table3.docx]

| **Primer** | **sequences (5' to 3')** |
| --- | --- |
| CTSG-F | GAGTCAGACGGAATCGAAACG |
| CTSG-R | CGGAGTGTATCTGTTCCCCTC |
| HKDC1-F | TGAGCCGTCTGACCAAAGC |
| HKDC1-R | TAGGGGTCGTCATAGGCACA |
| PTX3-F | CATCTCCTTGCGATTCTGTTTTG |
| PTX3-R | CCCATTCCGAGTGCTCCTGA |
| SPP1-F | CAGCCGTGGGAAGGACAGTTATG |
| SPP1-R | TCACATCGGAATGCTCATTGCTCTC |
