# Supplementary material for: Identification and validation of a prognostic signature comprising inflammation and pyroptosis-related genes in oral squamous cell carcinoma
Source: Front Immunol. 2026 Jul 7;17:1721849. doi: 10.3389/fimmu.2026.1721849 (PMC13384851; doi:10.3389/fimmu.2026.1721849)
Supplement: Supplementary file 17 [file Table4.docx]

**Table S8 Result of GO and KEGG Enrichment Analysis for IR&PRDEGs**

| ONTOLOGY | ID | Description | GeneRatio | BgRatio | pvalue | p.adjust | qvalue |
| --- | --- | --- | --- | --- | --- | --- | --- |
| BP | GO:0008217 | regulation of blood pressure | 8/51 | 187/18614 | 3.93E-08 | 8.13E-05 | 5.24E-05 |
| BP | GO:0019221 | cytokine-mediated signaling pathway | 10/51 | 492/18614 | 7.34E-07 | 4.21E-04 | 2.72E-04 |
| BP | GO:0006334 | nucleosome assembly | 6/51 | 118/18614 | 8.15E-07 | 4.21E-04 | 2.72E-04 |
| BP | GO:0051702 | biological process involved in interaction with symbiont | 6/51 | 118/18614 | 8.15E-07 | 4.21E-04 | 2.72E-04 |
| BP | GO:0000302 | response to reactive oxygen species | 7/51 | 205/18614 | 1.36E-06 | 4.50E-04 | 2.90E-04 |
| CC | GO:0062023 | collagen-containing extracellular matrix | 9/51 | 415/19518 | 1.12E-06 | 7.89E-05 | 6.06E-05 |
| CC | GO:0000786 | nucleosome | 6/51 | 133/19518 | 1.25E-06 | 7.89E-05 | 6.06E-05 |
| CC | GO:0044815 | DNA packaging complex | 6/51 | 164/19518 | 4.23E-06 | 1.78E-04 | 1.37E-04 |
| CC | GO:0032993 | protein-DNA complex | 6/51 | 225/19518 | 2.56E-05 | 8.08E-04 | 6.21E-04 |
| CC | GO:1904724 | tertiary granule lumen | 3/51 | 55/19518 | 4.01E-04 | 7.82E-03 | 6.01E-03 |
| MF | GO:0030527 | structural constituent of chromatin | 6/51 | 97/18369 | 2.76E-07 | 5.51E-05 | 3.95E-05 |
| MF | GO:0048018 | receptor ligand activity | 10/51 | 497/18369 | 9.07E-07 | 9.07E-05 | 6.49E-05 |
| MF | GO:0005125 | cytokine activity | 7/51 | 235/18369 | 3.68E-06 | 2.15E-04 | 1.54E-04 |
| MF | GO:0046982 | protein heterodimerization activity | 8/51 | 343/18369 | 4.31E-06 | 2.15E-04 | 1.54E-04 |
| MF | GO:0004252 | serine-type endopeptidase activity | 6/51 | 170/18369 | 7.34E-06 | 2.93E-04 | 2.10E-04 |
| KEGG | hsa05322 | Systemic lupus erythematosus | 9/41 | 137/8746 | 9.99E-09 | 1.32E-06 | 1.18E-06 |
| KEGG | hsa04613 | Neutrophil extracellular trap formation | 9/41 | 191/8746 | 1.79E-07 | 1.18E-05 | 1.06E-05 |
| KEGG | hsa05034 | Alcoholism | 8/41 | 188/8746 | 2.04E-06 | 9.00E-05 | 8.03E-05 |
| KEGG | hsa05202 | Transcriptional misregulation in cancer | 6/41 | 193/8746 | 2.52E-04 | 8.33E-03 | 7.44E-03 |

GO，Gene Ontology；BP，Biological Process；CC，Cellular Component；MF，Molecular Function；KEGG，Kyoto Encyclopedia of Genes and Genomes；IR&PRDEGs，Inflammatory-Related and Pyroptosis-Related Differentially Expressed Genes。
