# Supplementary material for: Identification and validation of a prognostic signature comprising inflammation and pyroptosis-related genes in oral squamous cell carcinoma
Source: Front Immunol. 2026 Jul 7;17:1721849. doi: 10.3389/fimmu.2026.1721849 (PMC13384851; doi:10.3389/fimmu.2026.1721849)
Supplement: Supplementary file 18 [file Table5.docx]

**Table S9 Results of GSEA for Risk Group**

| ID | setSize | EnrichmentScore | NES | pvalue | p.adjust | qvalue |
| --- | --- | --- | --- | --- | --- | --- |
| REACTOME_PRC2_METHYLATES_HISTONES_AND_DNA | 66 | 0.629457 | 2.105346 | 6.27E-07 | 2.79E-05 | 2.25E-05 |
| REACTOME_HDACS_DEACETYLATE_HISTONES | 86 | 0.603606 | 2.102158 | 7.64E-08 | 4.35E-06 | 3.52E-06 |
| REACTOME_HDMS_DEMETHYLATE_HISTONES | 47 | 0.534179 | 1.684003 | 0.002361 | 0.022331 | 0.018042 |
| REACTOME_OXIDATIVE_STRESS_INDUCED_SENESCENCE | 117 | 0.457577 | 1.668523 | 0.000297 | 0.004024 | 0.003251 |
| REACTOME_RMTS_METHYLATE_HISTONE_ARGININES | 72 | 0.49362 | 1.662464 | 0.002754 | 0.0252 | 0.02036 |
| REACTOME_HATS_ACETYLATE_HISTONES | 134 | 0.443164 | 1.655878 | 0.000274 | 0.003829 | 0.003093 |
| REACTOME_PRE_NOTCH_EXPRESSION_AND_PROCESSING | 103 | 0.410516 | 1.470802 | 0.006316 | 0.048658 | 0.039313 |
| REACTOME_MAPK_FAMILY_SIGNALING_CASCADES | 320 | -0.34716 | -1.45024 | 0.000896 | 0.010213 | 0.008251 |
| KEGG_MAPK_SIGNALING_PATHWAY | 265 | -0.3551 | -1.45653 | 0.001353 | 0.014492 | 0.011708 |
| REACTOME_PI3K_AKT_SIGNALING_IN_CANCER | 105 | -0.4426 | -1.61378 | 0.002689 | 0.024805 | 0.020041 |
| PID_IL4_2PATHWAY | 64 | -0.60152 | -2.02085 | 5.88E-06 | 0.000161 | 0.00013 |
| WP_INFLAMMATORY_BOWEL_DISEASE_SIGNALING | 42 | -0.65121 | -2.02146 | 4.31E-05 | 0.000799 | 0.000646 |
| PID_IL23_PATHWAY | 37 | -0.68354 | -2.06292 | 2.27E-05 | 0.000472 | 0.000381 |
| WP_INFLAMMATORY_RESPONSE_PATHWAY | 30 | -0.71652 | -2.08889 | 3.89E-05 | 0.00073 | 0.00059 |
| PID_IL12_STAT4_PATHWAY | 32 | -0.72721 | -2.17018 | 8.92E-06 | 0.000219 | 0.000177 |
| PID_IL12_2PATHWAY | 62 | -0.67412 | -2.26419 | 3.86E-08 | 2.48E-06 | 2E-06 |
| REACTOME_ANTI_INFLAMMATORY_RESPONSE_FAVOURING_LEISHMANIA_PARASITE_INFECTION | 133 | -0.78715 | -2.97883 | 1E-10 | 9.15E-09 | 7.39E-09 |
| REACTOME_FCGR3A_MEDIATED_IL10_SYNTHESIS | 94 | -0.87164 | -3.13173 | 1E-10 | 9.15E-09 | 7.39E-09 |
| REACTOME_FCERI_MEDIATED_MAPK_ACTIVATION | 87 | -0.88331 | -3.1459 | 1E-10 | 9.15E-09 | 7.39E-09 |
| REACTOME_FCERI_MEDIATED_NF_KB_ACTIVATION | 136 | -0.83483 | -3.18171 | 1E-10 | 9.15E-09 | 7.39E-09 |

GSEA，Gene Set Enrichment Analysis
