# Supplementary material for: Identification and validation of a prognostic signature comprising inflammation and pyroptosis-related genes in oral squamous cell carcinoma
Source: Front Immunol. 2026 Jul 7;17:1721849. doi: 10.3389/fimmu.2026.1721849 (PMC13384851; doi:10.3389/fimmu.2026.1721849)
Supplement: Supplementary file 19 [file Table6.docx]

**The interacting proteins of four model genes (Model Genes) and their functionally similar genes**

# Organism: H. sapiens

# Application version: 3.6.0

# Database version: 13 August 2021 00:00:00

# Network generated on: 10 April 2024

# Notes: Network weight reflects the data source relevance for predicting the function of interest

Entity 1 Entity 2 Weight Network group Network

ITGA5 PTX3 0.010019456 Co-expression Wang-Maris-2006

F2RL1 F2 0.012967881 Co-expression Wang-Maris-2006

PLAUR ITGA5 0.00916616 Co-expression Wang-Maris-2006

CD44 ITGA8 0.018626168 Co-expression Wang-Maris-2006

IL6 PLAUR 0.017355137 Co-expression Wang-Maris-2006

F2RL1 HKDC1 0.017614042 Co-expression Mallon-McKay-2013

ITGA4 PTX3 0.007722231 Co-expression Mallon-McKay-2013

ITGA4 CD44 0.012917398 Co-expression Mallon-McKay-2013

IL6 CTSG 0.017192122 Co-expression Mallon-McKay-2013

ITGA8 ITGA5 0.016019678 Co-expression Roth-Zlotnik-2006

F2 IGFALS 0.004538575 Co-expression Roth-Zlotnik-2006

HK3 CTSG 0.004790601 Co-expression Roth-Zlotnik-2006

PLAUR CTSG 0.008614674 Co-expression Roth-Zlotnik-2006

PLAUR HK3 0.011789582 Co-expression Roth-Zlotnik-2006

ITGA4 PTX3 0.013333287 Co-expression Roth-Zlotnik-2006

ITGA5 CTSG 0.013155637 Co-expression Ramaswamy-Golub-2001

IGFBP3 SPP1 0.018625723 Co-expression Ramaswamy-Golub-2001

PLAUR PTX3 0.01785493 Co-expression Ramaswamy-Golub-2001

CD44 ITGA5 0.020487975 Co-expression Ramaswamy-Golub-2001

IL6 PTX3 0.018672485 Co-expression Ramaswamy-Golub-2001

ITGA8 PTX3 0.011890879 Co-expression Innocenti-Brown-2011

PLAUR PTX3 0.0054756687 Co-expression Innocenti-Brown-2011

PLAUR HK2 0.00429759 Co-expression Innocenti-Brown-2011

CD44 HK2 0.0075117755 Co-expression Innocenti-Brown-2011

CD44 PLAUR 0.005102818 Co-expression Innocenti-Brown-2011

IL6 PTX3 0.00804187 Co-expression Innocenti-Brown-2011

IL6 PLAUR 0.004362823 Co-expression Innocenti-Brown-2011

ITGAV SPP1 0.016858555 Co-expression Innocenti-Brown-2011

IL6 CD44 0.014429327 Co-expression Alizadeh-Staudt-2000

CTSG HKDC1 0.014357841 Co-expression Dobbin-Giordano-2005

HK2 PTX3 0.017356426 Co-expression Dobbin-Giordano-2005

ITGA5 SPP1 0.017955024 Co-expression Bild-Nevins-2006 B

GCK ITGA8 0.0037051085 Co-expression Bild-Nevins-2006 B

GCK F2 0.004747455 Co-expression Bild-Nevins-2006 B

GCK ITGA9 0.006003605 Co-expression Bild-Nevins-2006 B

GCK F2RL1 0.0070196902 Co-expression Bild-Nevins-2006 B

PLAUR SPP1 0.026055178 Co-expression Bild-Nevins-2006 B

PLAUR ITGA5 0.01884279 Co-expression Bild-Nevins-2006 B

GP1BA ITGA9 0.018702097 Co-expression Bild-Nevins-2006 B

IGFALS ITGA5 0.015745455 Co-expression Burington-Shaughnessy-2008

GCK F2 0.005040421 Co-expression Burington-Shaughnessy-2008

SPP1 PTX3 0.012055541 Co-expression Arijs-Rutgeerts-2009

HK3 PTX3 0.012623857 Co-expression Arijs-Rutgeerts-2009

HK3 ITGA5 0.007569174 Co-expression Arijs-Rutgeerts-2009

HK2 HK1 0.006909457 Co-expression Arijs-Rutgeerts-2009

PLAUR HK1 0.0027120837 Co-expression Arijs-Rutgeerts-2009

PLAUR HK2 0.01080123 Co-expression Arijs-Rutgeerts-2009

IL6 PTX3 0.0111967325 Co-expression Arijs-Rutgeerts-2009

IL6 ITGA5 0.007166864 Co-expression Arijs-Rutgeerts-2009

IGFBP3 HK1 0.009569087 Co-expression Jiang-de Kok-2017

IL6 PTX3 0.0028733807 Co-expression Jiang-de Kok-2017

GP1BA IL6 0.004087493 Co-expression Jiang-de Kok-2017

REN IL6 0.0035721287 Co-expression Jiang-de Kok-2017

ITGAV ITGA5 0.007029102 Co-expression Perou-Botstein-2000

ITGAV PLAUR 0.00811855 Co-expression Perou-Botstein-2000

CD44 PLAUR 0.008982129 Co-expression Chen-Brown-2002

ITGAV SPP1 0.017366713 Co-expression Chen-Brown-2002

HK2 HK1 0.012255452 Co-expression Wang-Cheung-2015

PLAUR SPP1 0.0037277474 Co-expression Wang-Cheung-2015

ITGA5 CTSG 0.012709231 Co-expression Wu-Garvey-2007

ITGAV PTX3 0.008258504 Co-expression Wu-Garvey-2007

ITGA8 PTX3 0.0150701795 Co-expression Rosenwald-Staudt-2001

CD44 PLAUR 0.0055626193 Co-expression Ross-Perou-2001

IL6 IGFBP3 0.016392434 Co-expression Ross-Perou-2001

GP1BA CTSG 0.02104504 Co-localization Schadt-Shoemaker-2004

REN IGFBP3 0.009119114 Co-localization Schadt-Shoemaker-2004

ITGAV IL6 0.011209603 Co-localization Schadt-Shoemaker-2004

CTSG PTX3 0.006695612 Co-localization Johnson-Shoemaker-2003

GP1BA HK2 0.022366172 Co-localization Johnson-Shoemaker-2003

ITGAV SPP1 0.01939103 Co-localization Johnson-Shoemaker-2003

F2 SPP1 0.76536685 Co-localization Chen-Huang-2014

IGFALS ITGA8 0.0018836893 Genetic Interactions Lin-Smith-2010

ITGA9 CTSG 0.0022959346 Genetic Interactions Lin-Smith-2010

ITGA9 SPP1 0.00052351964 Genetic Interactions Lin-Smith-2010

ITGA9 ITGA8 0.0006864435 Genetic Interactions Lin-Smith-2010

F2RL1 SPP1 0.0019398034 Genetic Interactions Lin-Smith-2010

IGFBP3 ITGA8 0.0011630048 Genetic Interactions Lin-Smith-2010

HK2 HK1 0.0037245788 Genetic Interactions Lin-Smith-2010

CD44 ITGA8 0.0009535509 Genetic Interactions Lin-Smith-2010

IL6 CD44 0.0030648613 Genetic Interactions Lin-Smith-2010

ATP6AP2 SPP1 0.0003998308 Genetic Interactions Lin-Smith-2010

ATP6AP2 ITGA9 0.00042279437 Genetic Interactions Lin-Smith-2010

ATP6AP2 PLAUR 0.001016713 Genetic Interactions Lin-Smith-2010

ITGA5 SPP1 0.018451687 Pathway Wu-Stein-2010

ITGA8 SPP1 0.018455131 Pathway Wu-Stein-2010

ITGA8 ITGA5 0.0065504787 Pathway Wu-Stein-2010

F2 IGFALS 0.022198165 Pathway Wu-Stein-2010

ITGA9 SPP1 0.020077987 Pathway Wu-Stein-2010

ITGA9 ITGA8 0.007127827 Pathway Wu-Stein-2010

F2RL1 CTSG 0.31954107 Pathway Wu-Stein-2010

F2RL1 F2 0.034152385 Pathway Wu-Stein-2010

IGFBP3 IGFALS 0.093437456 Pathway Wu-Stein-2010

IGFBP3 F2 0.02459752 Pathway Wu-Stein-2010

ITGA4 SPP1 0.017122433 Pathway Wu-Stein-2010

ITGA4 ITGA8 0.006078585 Pathway Wu-Stein-2010

GP1BA F2 0.026311407 Pathway Wu-Stein-2010

ITGAV SPP1 0.014184197 Pathway Wu-Stein-2010

ITGAV ITGA8 0.0050354907 Pathway Wu-Stein-2010

ITGA8 SPP1 0.1558835 Pathway NCI_NATURE

ITGA9 SPP1 0.05936842 Pathway NCI_NATURE

PLAUR CTSG 0.3194061 Pathway NCI_NATURE

PLAUR ITGA5 0.03378003 Pathway NCI_NATURE

ITGA4 SPP1 0.03893785 Pathway NCI_NATURE

ITGA4 CD44 0.0843568 Pathway NCI_NATURE

ITGAV SPP1 0.023089461 Pathway NCI_NATURE

ITGAV PLAUR 0.009681994 Pathway NCI_NATURE

ITGA5 SPP1 1 Pathway REACTOME

IGFALS CTSG 0.48993602 Pathway REACTOME

F2 IGFALS 0.09377939 Pathway REACTOME

IGFBP3 IGFALS 0.21014853 Pathway REACTOME

GP1BA F2 0.08974387 Pathway REACTOME

ITGA5 SPP1 0.0856682 Physical Interactions IREF-reactome

ITGA8 SPP1 0.20388184 Physical Interactions IREF-reactome

IGFALS CTSG 0.10776068 Physical Interactions IREF-reactome

F2 IGFALS 0.018921247 Physical Interactions IREF-reactome

ITGA9 SPP1 0.17291312 Physical Interactions IREF-reactome

F2RL1 F2 0.00697005 Physical Interactions IREF-reactome

IGFBP3 CTSG 0.15013216 Physical Interactions IREF-reactome

IGFBP3 IGFALS 0.11752498 Physical Interactions IREF-reactome

IGFBP3 F2 0.026361078 Physical Interactions IREF-reactome

GCK HK3 0.054854985 Physical Interactions IREF-reactome

GCK HK1 0.054854985 Physical Interactions IREF-reactome

GCK HK2 0.054854985 Physical Interactions IREF-reactome

ACE2 CTSG 0.1293132 Physical Interactions IREF-reactome

CD44 SPP1 0.10652069 Physical Interactions IREF-reactome

ITGA4 SPP1 0.09118143 Physical Interactions IREF-reactome

ATP6AP2 CTSG 0.10399382 Physical Interactions IREF-reactome

ATP6AP2 ACE2 0.09768918 Physical Interactions IREF-reactome

GP1BA F2 0.0151845645 Physical Interactions IREF-reactome

REN CTSG 0.10399382 Physical Interactions IREF-reactome

REN ACE2 0.09768918 Physical Interactions IREF-reactome

REN ATP6AP2 0.078561746 Physical Interactions IREF-reactome

ITGAV SPP1 0.04643321 Physical Interactions IREF-reactome

ITGA5 SPP1 0.0856682 Physical Interactions Vastrik-Stein-2007

ITGA8 SPP1 0.20388184 Physical Interactions Vastrik-Stein-2007

IGFALS CTSG 0.10776068 Physical Interactions Vastrik-Stein-2007

F2 IGFALS 0.018921247 Physical Interactions Vastrik-Stein-2007

ITGA9 SPP1 0.17291312 Physical Interactions Vastrik-Stein-2007

F2RL1 F2 0.00697005 Physical Interactions Vastrik-Stein-2007

IGFBP3 CTSG 0.15013216 Physical Interactions Vastrik-Stein-2007

IGFBP3 IGFALS 0.11752498 Physical Interactions Vastrik-Stein-2007

IGFBP3 F2 0.026361078 Physical Interactions Vastrik-Stein-2007

GCK HK3 0.054854985 Physical Interactions Vastrik-Stein-2007

GCK HK1 0.054854985 Physical Interactions Vastrik-Stein-2007

GCK HK2 0.054854985 Physical Interactions Vastrik-Stein-2007

ACE2 CTSG 0.1293132 Physical Interactions Vastrik-Stein-2007

CD44 SPP1 0.10652069 Physical Interactions Vastrik-Stein-2007

ITGA4 SPP1 0.09118143 Physical Interactions Vastrik-Stein-2007

ATP6AP2 CTSG 0.10399382 Physical Interactions Vastrik-Stein-2007

ATP6AP2 ACE2 0.09768918 Physical Interactions Vastrik-Stein-2007

GP1BA F2 0.0151845645 Physical Interactions Vastrik-Stein-2007

REN CTSG 0.10399382 Physical Interactions Vastrik-Stein-2007

REN ACE2 0.09768918 Physical Interactions Vastrik-Stein-2007

REN ATP6AP2 0.078561746 Physical Interactions Vastrik-Stein-2007

ITGAV SPP1 0.04643321 Physical Interactions Vastrik-Stein-2007

GP1BA F2 0.21437077 Physical Interactions IREF-quickgo

ITGA4 CD44 0.30671895 Physical Interactions IREF-dip

CD44 IGFBP3 1 Physical Interactions IREF-uniprotpp

IGFBP3 IGFALS 0.15103392 Physical Interactions BIOGRID-SMALL-SCALE-STUDIES

CD44 IGFBP3 0.022622067 Physical Interactions BIOGRID-SMALL-SCALE-STUDIES

REN ATP6AP2 0.52043957 Physical Interactions BIOGRID-SMALL-SCALE-STUDIES

ITGAV SPP1 0.009139864 Physical Interactions BIOGRID-SMALL-SCALE-STUDIES

ITGAV ITGA4 0.08257013 Physical Interactions BIOGRID-SMALL-SCALE-STUDIES

HK3 HKDC1 0.3105377 Physical Interactions Huttlin-Harper-2017

HK1 HKDC1 0.37878132 Physical Interactions Huttlin-Harper-2017

HK1 HK3 0.12672427 Physical Interactions Huttlin-Harper-2017

HK2 HK3 0.3105377 Physical Interactions Huttlin-Harper-2017

HK2 HK1 0.37878132 Physical Interactions Huttlin-Harper-2017

GP1BA CTSG 0.6731135 Physical Interactions Rual-Vidal-2005

F2 SPP1 0.05464521 Physical Interactions IREF-matrixdb

CD44 SPP1 0.07532241 Physical Interactions IREF-matrixdb

ITGA5 SPP1 0.41324386 Physical Interactions Lim-Zoghbi-2006

CD44 SPP1 0.23788252 Physical Interactions Lim-Zoghbi-2006

ITGAV SPP1 0.41324386 Physical Interactions Lim-Zoghbi-2006

ITGA5 SPP1 0.16565165 Physical Interactions IREF-spike

CD44 SPP1 0.103666626 Physical Interactions IREF-spike

GP1BA CTSG 0.7270887 Physical Interactions IREF-spike

ITGAV SPP1 0.33633593 Physical Interactions IREF-spike

F2 SPP1 0.025406528 Physical Interactions IREF-biogrid

HK3 HKDC1 0.32665792 Physical Interactions IREF-biogrid

HK1 HKDC1 0.08708621 Physical Interactions IREF-biogrid

HK1 HK3 0.028381627 Physical Interactions IREF-biogrid

IGFBP3 IGFALS 0.32067502 Physical Interactions IREF-biogrid

HK2 HK3 0.056947507 Physical Interactions IREF-biogrid

HK2 HK1 0.015182066 Physical Interactions IREF-biogrid

ITGA4 HK2 0.0062802667 Physical Interactions IREF-biogrid

REN ATP6AP2 0.052676566 Physical Interactions IREF-biogrid

HK3 HKDC1 0.28349328 Predicted Wu-Stein-2010

HK1 HKDC1 0.20183355 Predicted Wu-Stein-2010

HK1 HK3 0.20183355 Predicted Wu-Stein-2010

HK2 HKDC1 0.23013395 Predicted Wu-Stein-2010

HK2 HK3 0.23013395 Predicted Wu-Stein-2010

HK2 HK1 0.16384427 Predicted Wu-Stein-2010

GCK HKDC1 0.20321098 Predicted Wu-Stein-2010

GCK HK3 0.20321098 Predicted Wu-Stein-2010

GCK HK1 0.14467642 Predicted Wu-Stein-2010

GCK HK2 0.16496244 Predicted Wu-Stein-2010

CD44 SPP1 0.06923385 Predicted Wu-Stein-2010

CD44 IGFBP3 0.061302494 Predicted Wu-Stein-2010

ITGA4 ITGA5 0.108371496 Predicted Wu-Stein-2010

IL6 PTX3 0.44417873 Predicted Wu-Stein-2010

GP1BA CTSG 0.20004156 Predicted Wu-Stein-2010

REN ATP6AP2 0.26276293 Predicted Wu-Stein-2010

ITGA8 ITGA5 0.061651736 Shared protein domains INTERPRO

F2 CTSG 0.01601124 Shared protein domains INTERPRO

ITGA9 ITGA5 0.061651736 Shared protein domains INTERPRO

ITGA9 ITGA8 0.061651736 Shared protein domains INTERPRO

HK3 HKDC1 0.10470211 Shared protein domains INTERPRO

HK1 HKDC1 0.10470211 Shared protein domains INTERPRO

HK1 HK3 0.10470211 Shared protein domains INTERPRO

HK2 HKDC1 0.10470211 Shared protein domains INTERPRO

HK2 HK3 0.10470211 Shared protein domains INTERPRO

HK2 HK1 0.10470211 Shared protein domains INTERPRO

GCK HKDC1 0.10470211 Shared protein domains INTERPRO

GCK HK3 0.10470211 Shared protein domains INTERPRO

GCK HK1 0.10470211 Shared protein domains INTERPRO

GCK HK2 0.10470211 Shared protein domains INTERPRO

ITGA4 ITGA5 0.061651736 Shared protein domains INTERPRO

ITGA4 ITGA8 0.061651736 Shared protein domains INTERPRO

ITGA4 ITGA9 0.061651736 Shared protein domains INTERPRO

GP1BA IGFALS 0.013438895 Shared protein domains INTERPRO

ITGAV ITGA5 0.061651736 Shared protein domains INTERPRO

ITGAV ITGA8 0.061651736 Shared protein domains INTERPRO

ITGAV ITGA9 0.061651736 Shared protein domains INTERPRO

ITGAV ITGA4 0.061651736 Shared protein domains INTERPRO

ITGA8 ITGA5 0.05782498 Shared protein domains PFAM

F2 CTSG 0.0075720036 Shared protein domains PFAM

ITGA9 ITGA5 0.06903703 Shared protein domains PFAM

ITGA9 ITGA8 0.05782498 Shared protein domains PFAM

HK3 HKDC1 0.25 Shared protein domains PFAM

HK1 HKDC1 0.25 Shared protein domains PFAM

HK1 HK3 0.25 Shared protein domains PFAM

HK2 HKDC1 0.25 Shared protein domains PFAM

HK2 HK3 0.25 Shared protein domains PFAM

HK2 HK1 0.25 Shared protein domains PFAM

GCK HKDC1 0.25 Shared protein domains PFAM

GCK HK3 0.25 Shared protein domains PFAM

GCK HK1 0.25 Shared protein domains PFAM

GCK HK2 0.25 Shared protein domains PFAM

ITGA4 ITGA5 0.06903703 Shared protein domains PFAM

ITGA4 ITGA8 0.05782498 Shared protein domains PFAM

ITGA4 ITGA9 0.06903703 Shared protein domains PFAM

GP1BA IGFALS 0.017377699 Shared protein domains PFAM

ITGAV ITGA5 0.05782498 Shared protein domains PFAM

ITGAV ITGA8 0.077884085 Shared protein domains PFAM

ITGAV ITGA9 0.05782498 Shared protein domains PFAM

ITGAV ITGA4 0.05782498 Shared protein domains PFAM
